# Mechanism of Ribonuclease III Catalytic Regulation by Serine Phosphorylation

**DOI:** 10.1038/srep25448

**Published:** 2016-05-06

**Authors:** Swapna Gone, Mercedes Alfonso-Prieto, Samridhdi Paudyal, Allen W. Nicholson

**Affiliations:** 1 Department of Chemistry, Philadelphia PA, 19122, USA; 2 Institute for Computational Molecular Science, Philadelphia PA, 19122, USA; 3 Department of Biology, Temple University, Philadelphia PA, 19122, USA

## Abstract

Ribonuclease III (RNase III) is a conserved, gene-regulatory bacterial endonuclease that cleaves double-helical structures in diverse coding and noncoding RNAs. RNase III is subject to multiple levels of control, reflective of its global regulatory functions. *Escherichia coli* (*Ec*) RNase III catalytic activity is known to increase during bacteriophage T7 infection, reflecting the expression of the phage-encoded protein kinase, T7PK. However, the mechanism of catalytic enhancement is unknown. This study shows that *Ec*-RNase III is phosphorylated on serine *in vitro* by purified T7PK, and identifies the targets as Ser33 and Ser34 in the N-terminal catalytic domain. Kinetic experiments reveal a 5-fold increase in k_cat_ and a 1.4-fold decrease in K_m_ following phosphorylation, providing a 7.4–fold increase in catalytic efficiency. Phosphorylation does not change the rate of substrate cleavage under single-turnover conditions, indicating that phosphorylation enhances product release, which also is the rate-limiting step in the steady-state. Molecular dynamics simulations provide a mechanism for facilitated product release, in which the Ser33 phosphomonoester forms a salt bridge with the Arg95 guanidinium group, thereby weakening RNase III engagement of product. The simulations also show why glutamic acid substitution at either serine does not confer enhancement, thus underscoring the specific requirement for a phosphomonoester.

RNA maturation and decay pathways are fundamentally involved in gene expression and regulation in bacterial cells, and are defined by the coordinated action of endoribonucleases and exoribonucleases. The nucleases act in conjunction with other factors, including small noncoding RNAs, and are subject to multiple levels of regulation in response to stress and other external cues^1^,^2^,^3^. As such, ribonucleases provide central points of control over post-transcriptional network function. Ribonuclease regulation of RNA function, and the mechanisms of action of ribonuclease regulators are not well understood, but are receiving increased scrutiny as potential points of drug intervention^4^,^5^.

Ribonuclease III is a conserved bacterial endonuclease that site-specifically cleaves double-stranded(ds) structures in diverse cellular, plasmid and phage RNAs^6^,^7^. The RNase III polypeptide (~220 amino acids) consists of an N-terminal catalytic domain [RIIID; ~150 amino acids (aa)] and a C-terminal dsRNA-binding domain (dsRBD; ~65 aa) joined by a short (10 aa) flexible linker. The active form of the enzyme is a homodimer, with a functionally independent catalytic site in each subunit and two dsRBDs that assist in substrate binding. The catalytic sites employ Mg^2+^ ions to hydrolyze phosphodiesters, providing products with two-nucleotide, 3′-overhangs and 5′-phosphomonoester, 3′-hydroxyl termini^6^,^7^. A primary substrate for RNase III is the ~5500 nt transcript of the rRNA operons, containing the 16S, 23S and 5S rRNAs, with the enzyme acting co-transcriptionally to provide the immediate precursors to the mature rRNAs^8^. RNase III also can determine mRNA half-life by catalyzing the rate-limiting cleavage step in the decay pathway^9^,^10^,^11^. Double-helical structures that are formed by binding of small noncoding RNAs (sRNAs) provide RNase III targets, and regulate mRNA translation and/or stability^12^,^13^. The diversity of RNase III targets and the multiple actions of the enzyme in conjunction with sRNAs and other factors underscore the global regulatory function of RNase III^6^,^7^,^14^.

A number of bacteriophages employ RNase III in their infection strategies. The coliphage T7 expresses multiple transcripts whose maturation involves RNase III action. Immediately following T7 infection of *Escherichia coli*, the host RNA polymerase transcribes the phage early region, generating a ~7,000 nt polycistronic mRNA precursor that is processed co-transcriptionally by RNase III at five sites to provide the mature, optimally functional mono- and dicistronic mRNAs^15^,^16^. One of the early mRNAs encodes the single-subunit RNA polymerase that transcribes the T7 middle and late genes, yielding large amounts of mRNA precursors that also are processed by RNase III^16^. While RNase III is not essential for T7 growth under standard growth conditions, it is important for optimal translation of at least several phage mRNAs^17^. RNase III is a low abundance enzyme^11^, and no new RNase III is synthesized during T7 infection since host transcription is suppressed. Thus, the copious production of T7 mRNAs is expected to place a premium on the ability of the existing RNase III population to function at maximum efficiency.

The T7 0.7 early gene encodes a ~40 kDa polypeptide that has an N-terminal domain (~30 kDa) possessing an ATP-dependent, serine/threonine-specific protein phosphotransferase activity (T7PK), and a C-terminal domain (~10 kDa) that exerts host transcription shut-off^18^,^19^,^20^. Expression of T7PK results in the phosphorylation of multiple cellular proteins, including translation factors and the β ′ subunit of the host RNA polymerase^21^,^22^,^23^. Phosphorylation of the β ′ subunit renders RNA polymerase responsive to otherwise weak transcription terminator signals, thereby attenuating host gene expression^23^. T7PK undergoes self-phosphorylation on serine, which downregulates its catalytic activity^19^,^20^,^24^. T7PK is not essential for T7 growth under normal laboratory conditions, but is practically essential at elevated temperatures or during carbon/energy limitation^25^. The T7PK-dependent modification of components of the gene expression machinery suggests a primary role of T7PK in redirecting gene expression from host to phage as part of the infection strategy.

Several *E. coli* enzymes involved in T7 RNA metabolism are modified during infection. The phosphorylation of RNA degradosome components RNase E and RNA helicase RhlB serves to stabilize the mRNAs synthesized by the T7 RNA polymerase^26^,^27^. RNase III also is phosphorylated during T7 infection in a T7PK-dependent manner, and the catalytic activity of the enzyme increases ~4–fold upon phosphorylation *in vivo*^28^. Serine was identified as the *in vivo* target; however, the location of the serine(s) was not determined, and how phosphorylation enhances catalytic activity was not defined. We present here an analysis of T7PK phosphorylation of RNase III, and describe the structural basis for the catalytic enhancement of dsRNA processing by phosphorylation.

## Results and Discussion

### T7PK phosphorylates serine in the α_2_-α_3_ loop of the RNase III catalytic domain

**Figure 1:**
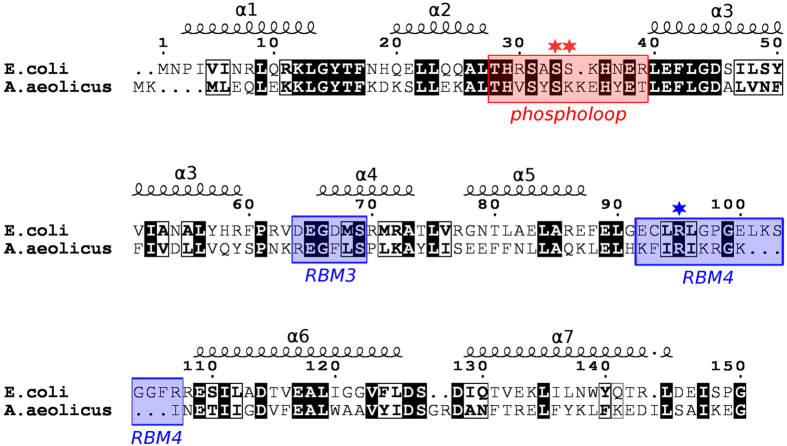
Alignment of *Escherichia coli* (*Ec*-)RNase III and *Aquifex aeolicus* (*Aa*-)RNase III sequences. Only the catalytic domains (RIIID) of the *Ec*-RNase III and *Aa*-RNase III polypeptides are shown. Black highlighted residues indicate conservation and the boxed residues indicate chemical similarity. The secondary structural elements of *Aa*-RNase III are shown on top. The segment highlighted in red (*phospholoop*) corresponds to the loop connecting the α _2_ and α _3_ helices, and contains the serine targets of T7PK (see Results and Discussion). The regions highlighted in blue correspond to the RNA-binding motifs 3 and 4 (RBM3 and RBM4) as described elsewhere^37^. Supplementary Figure S1 provides the alignment of complete RNase III polypeptide sequences.

**Figure 2:**
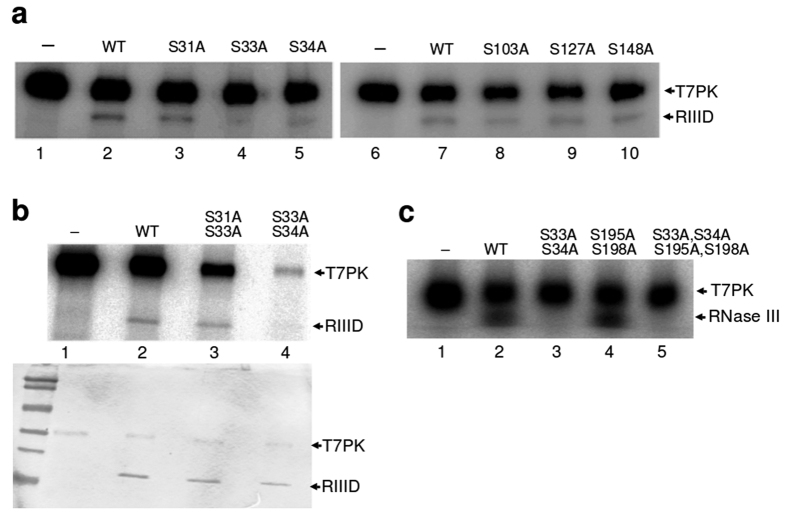
Alanine mutation identifies Ser33 and Ser34 as *in vitro* targets of *Ec*-RNase III phosphorylation by T7PK. H6-tagged Ec-RIIID polypeptides with the indicated alanine mutations were purified in soluble form and subjected to phosphorylation *in vitro* using dephosphorylated T7PK and [γ -^32^P]ATP (see Materials and methods). The concentration of Ec-RIIID (or mutant) or RNase III (or mutant) was 2.5 μ M and dephosphorylated T7PK was 3.3 μ M. Aliquots were electrophoretically fractionated by SDS-PAGE, and removal of unincorporated radioactivity accomplished by gel staining and destaining (see also Materials and methods). Reactions were imaged by phosphorimaging. **(a)** Effect of single alanine mutations on H6-*Ec*-RIIID phosphorylation. Positions of (self-phosphorylated) T7PK and RIIID are indicated on the right. The first lane in each gel image displays a control reaction where RIIID was omitted. **(b)** Effect of double alanine mutations on H6-*Ec*-RIIID phosphorylation. The upper image is the phosphorimage of phosphorylation reactions involving RIIID with double alanine mutations in the phospholoop. The lower image is the corresponding Coomassie-stained gel image showing the locations of the T7PK and RIIID polypeptides. **(c)** Effect of double and quadruple alanine mutations on T7PK phosphorylation of *Ec*-RNase III. S195 and S198 are surface serine residues in the C-terminal dsRBD.

The observations that *E. coli* (*Ec*) RNase III is phosphorylated on serine in T7-infected cells^22^,^28^, and that the N-terminal catalytic domain (RIIID) (encompassing residues 1–150) is phosphorylated by T7PK *in vitro*^24^ afforded a strategy for identifying the phosphorylation site(s), using the RIIID polypeptide as the target for selective mutation. The *Ec*-RIIID polypeptide contains ten serines (Fig. 1). Based on a homology-modeled *Ec*-RNase III structure^29^, six of the ten serines (Ser31, 33, 34, 103, 127 and 148) are located on the protein surface (Suppl. Figs S1 and S2), and were chosen as targets for alanine mutation. The proximity of S31, S33 and S34 in the loop connecting the α _2_ and α _3_ helices (Fig. 1) also prompted the creation of double mutations within the loop. The mutant RIIID polypeptides were purified as soluble, N-terminal hexahistidine (H6)-tagged species and *in vitro* phosphorylation assays were performed as described (see Materials and methods) using purified recombinant T7PK^24^. Assays of the S31A, S33A, S34A, S103A, S127A, and S148A mutants are shown in Fig. 2. The reaction involving RIIID (Fig. 2a, lanes 2 and 7) contains a ^32^P-labeled species that migrates with a mass commensurate with the size of the H6-tagged RIIID polypeptide (~20 kDa), and confirms that RIIID is a substrate for T7PK, as reported elsewhere^24^. Self-phosphorylated T7PK (~30 kDa) is the second, slower-migrating species^24^. The S31A, S103A, S127A and S148A RIIID mutants are phosphorylated to a comparable extent as RIIID (Fig. 2a, lanes 3, and 8–10). In contrast, the S33A and S34A mutants exhibit strongly reduced phosphorylation (Fig. 2a, compare lanes 4 and 5 with lane 2), while the S33A, S34A double mutant is essentially fully resistant to phosphorylation (Fig. 2b, compare lane 4 with lane 2; see also Table 1).

In order to rule out any indirect effect of the C-terminal dsRBD on phosphorylation we also assessed the impact of the S33A, S34A double mutation on phosphorylation of full-length enzyme. Since the electrophoretic mobilities of T7PK and RNase III are similar, a quantitative analysis was not possible. However, the S33A,S34A double mutation also suppresses phosphorylation of the RNase III polypeptide (Fig. 2c, compare lanes 3 and 5 with lane 2). We conclude that (i) S33 and S34 are targets in the full-length enzyme, and (ii) the dsRBD is not a phosphorylation target. In this regard, alanine mutation of the two surface-located serines in the dsRBD (S195 and S198) (see Suppl. Figs S1 and S2) did not alter phosphorylation (Fig. 2c, lane 4). Interestingly, the RIIID S31A, S33A mutant is phosphorylated to a comparable extent as RIIID (Fig. 2b, compare lanes 2 and 3). The enhancement of S34 phosphorylation by the S31A mutation may reflect a conformational change in the α _2_–α _3_ loop that alters the T7PK-RNase III interaction (see also below). T7PK self-phosphorylation is significantly reduced in the reaction involving the S33A, S34A RIIID double mutant (Fig. 2b, compare lanes 4 and 2), and to a lesser extent with S34A (Fig. 2a, compare lanes 5 and 2). The reduction is not due to differing amounts of protein (see lower figure in Fig. 2b), and may reflect formation of a stable, unreactive complex of T7PK and the double mutant protein.

While the mutational approach shows that the α _2_-α _3_ loop containing S33 and S34 (hereafter termed the phospholoop – see Fig. 1) is the target for T7PK action, it cannot distinguish between the possibilities that (i) S33 and S34 are phosphorylated in a stochastic, mutually exclusive manner, or (ii) that only one of the two serines is the target, with the other serine important for target recognition and/or reactivity. Thus, several attempts were made to map the *Ec*-RNase III phosphorylation site(s) by mass spectrometric analysis of enzymatically-generated peptides. However, the analyses were inconclusive, as the tryptic pentapeptide [(R)SA**SS**K_35_] containing S33 and S34 (underlined boldface) was the only member of the total set of tryptic peptides that was not represented, in either phosphorylated or unphosphorylated form, in a coverage map of the *Ec*-RNase III polypeptide (S. G. and A.W.N., unpublished results). The failure may reflect the short length of the peptide; however, attempts to map the site by using mutation to create longer tryptic peptides also were unsuccessful (S.G. and A.W.N., unpublished results).

The stoichiometry of RIIID phosphorylation was determined by direct radiometric measurement of the ^32^P-labeled polypeptide in excised polyacrylamide gel slices (see Materials and methods). The analysis reveals 0.32 mole phosphate incorporated per mole RIIID homodimer (Table 1). In contrast, the S33A,S34A RIIID double mutant exhibited a level of phosphorylation no greater than the background value (0.04 mol phosphate per mol protein). Additional treatment of phosphorylated RIIID with fresh T7PK and ATP did not increase the level of incorporation, indicating that a fraction of RIIID is resistant to phosphorylation.

### Phosphorylation increases the k_cat_ for RNase III cleavage of substrate

**Figure 3:**
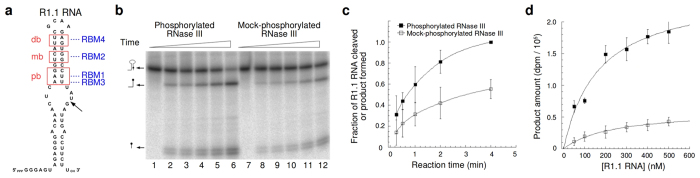
RNase III phosphorylation stimulates R1.1 RNA cleavage *in vitro*. **(a)** Sequence and secondary structure of R1.1 RNA. The positions of protein interaction (pb, proximal box; mb, middle box; and db, distal box) are highlighted with red boxes. The interacting protein domains [RNA-binding motifs (RBMs) 1–4] are indicated in blue. The arrow marks the site of RNase III cleavage. **(b)** Gel phosphorimage of time-course assays of cleavage of internally-^32^P-labeled R1.1 RNA (200 nM) by *Ec*-RNase III (20 nM), in phosphorylated or nonphosphorylated form. Lanes 1–6 shows a representative time course assay involving phosphorylated *Ec*-RNase III, while lanes 7–12 show the time course assay involving mock-phosphorylated enzyme. Lanes 2–6 and 8–12 show 15 sec, 30 sec, 1 min, 2 min and 4 min reaction time points; lanes 1 and 7 represent control reactions where R1.1 RNA was incubated for 1 min in an otherwise complete reaction, but lacking MgCl_2_. The RNA doublets at the bottom of the lanes are R1.1 RNA 3′-end-containing products, the longer product of which contains an additional non-templated nucleotide incorporated during R1.1 RNA synthesis. **(c)** Graphic depiction of time course reactions of R1.1 RNA cleavage by phosphorylated and mock-phosphorylated *Ec*-RNase III. The points are the average of two experiments, with maximum errors shown. **(d)** Substrate concentration dependence of the initial rate of cleavage of R1.1 RNA by phosphorylated and mock-phosphorylated *Ec*-RNase III. Cleavage reactions involved 10 nM *Ec*-RNase III, and the indicated concentrations of internally ^32^P-labeled R1.1 RNA. Reactions were performed in duplicate. The kinetic constants are provided in Table 2.

To determine whether phosphorylation enhances *Ec*-RNase III catalytic activity *in vitro* we carried out a kinetic analysis of phosphorylated and unphosphorylated enzyme. The phosphorylation reaction conditions allowed maximal covalent modification (~0.32 mol phosphate per RNase III homodimer – see Table 1). The control (mock phosphorylation) reaction contained all components except for Mg^2+^ ion, which is required for T7PK catalytic activity^19^,^20^. As a control, the same kinetic analysis was performed for the S33A, S34A mutant, which is fully resistant to phosphorylation (see Fig. 2b and Table 1). The treated enzymes were purified (see Materials and methods) and employed in multiple-turnover cleavage assays using internally ^32^P-labeled R1.1 RNA as substrate. R1.1 RNA (Fig. 3a) has been used in previous enzyme kinetic analyses^30^,^31^,^32^ and is cleaved by *Ec*-RNase III at a single phosphodiester, forming 47 nt and 13 nt products. A time course assay of R1.1 RNA cleavage (Fig. 3b,c) shows that phosphorylated RNase III cleaves substrate more rapidly than mock-phosphorylated enzyme. Moreover, the catalytic action of the S33A, S34A double mutant (Suppl. Fig. S3) is not increased by T7PK treatment, thus providing additional support that the α _2_-α _3_ loop is the phosphorylation target. The steady-state parameters k_cat_ and K_m_ (Table 2) were determined from the data in Figure 3d, and show that phosphorylated RNase III exhibits a 5–fold greater k_cat_ and a 1.4–fold lower K_m_ compared to unphosphorylated enzyme. Together, the values provide a 7.4–fold increase in catalytic efficiency (k_cat_/K_m_). Since only ~30% phosphorylation of RNase III was achieved, the 7.4–fold increase most likely represents a lower limit to catalytic efficiency enhancement.

A gel mobility shift assay was used to assess whether phosphorylation alters substrate affinity. In this assay Mg^2+^ was replaced by Ca^2+^, which prevents phosphodiester cleavage while supporting substrate binding^33^. Single complexes with similar mobilities were observed for phosphorylated and mock-phosphorylated enzyme, and complex formation exhibited closely similar concentration dependencies (Suppl. Fig. S4). This result is consistent with the minor change in K_m_ (see above) and provides further support that phosphorylation primarily affects one or more events following substrate binding.

### Evidence for enhanced product release and specific requirement for a phosphomonoester

A proposed kinetic scheme for RNase III involves (i) substrate recognition by the dsRBD; (ii) substrate engagement by the RIIID; (iii) phosphodiester hydrolysis; and (iv) product release^6^,^7^. Based on the gel shift assay results (Suppl. Fig. S4) and only a minor change in K_m_ (Table 2), an effect of phosphorylation on step (i) or (ii) can be ruled out. The increase in k_cat_, however, suggests a change in the rate of phosphodiester hydrolysis (iii) and/or product release (iv). To differentiate between these two possibilities, time-course assays of R1.1 RNA cleavage were performed under single-turnover (enzyme excess) conditions, such that the measured rate (described by a single exponential decay constant, k_2_) would only reflect the hydrolytic step (see Suppl. Fig. S5). The calculated k_2_ values (Table 2) show that there is no significant difference in the rate of R1.1 RNA cleavage by phosphorylated and mock-phosphorylated *Ec*-RNase III. Based on the increased k_cat_, but unaltered k_2_ (Table 2), we conclude that the step responsive to phosphorylation is product release.

To determine whether the rate enhancement reflects introduction of negative charge in the α _2_-α _3_ phospholoop, the catalytic activity of the S33E, S34E double mutant enzyme was compared with wild-type enzyme. Similar to a serine phosphomonoester at pH 7.5, two glutamic acid residues would provide two negative charges^34^,^35^. If negative charge is the functionally important factor, then the double mutant protein would exhibit enhanced activity compared to the normal enzyme. In a time-course assay under conditions of excess substrate the S33E, S34E mutant cleaved R1.1 RNA with an efficiency comparable to, but not greater than unphosphorylated RNase III (S.G. and A.W.N., data not shown). We conclude that the catalytic enhancement reflects a specific requirement for a phosphomonoester group in the α _2_-α _3_ phospholoop, rather than negative charge *per se*.

### Computational approaches suggest a structural model for phosphorylation dependent catalytic enhancement

**Figure 4:**
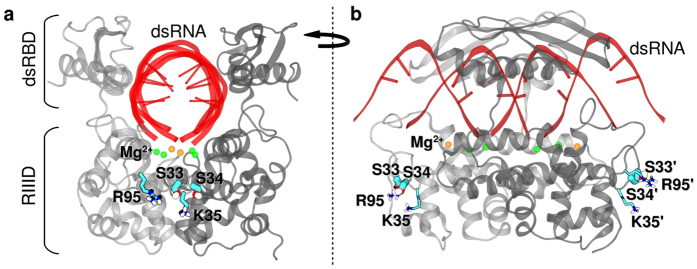
*Ec*-RNase III homology-modeled structure in complex with cleaved dsRNA. The protein is represented in gray cartoon form, and the cleaved dsRNA in red. Residues S33, S34, K35 and R95 are shown as licorice with C, O and N atoms in cyan, red and blue, respectively; only polar H atoms (white) are displayed. The two catalytic Mg^2+^ ions are shown as green spheres, whereas the third Mg^2+^ ion, which has been proposed to be involved in product release^46^, is in orange. **(a)** Front view of the structure, along the axis of the cleaved dsRNA. The two subunits of the homodimer are represented in light and dark gray cartoon, respectively. The two domains of homodimeric RNase III (RIIID and dsRBD) are indicated. **(b)** Side view of the structure, upon 90 degree rotation along the axis perpendicular to the cleaved dsRNA. The location of residues S33, S34, K35 and R95 (and their symmetric counterparts S33′, S34′, K35′ and R95′ in the other subunit of the homodimer) is shown in licorice representation.

**Figure 5:**
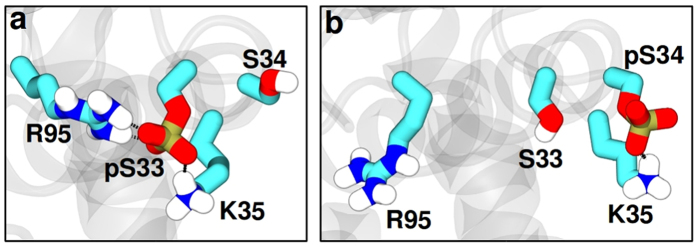
Representative interactions of the phosphorylated serine residues. **(a)** pS33 and **(b)** pS34 observed in the molecular dynamics simulations of *Ec*-RNase III in complex with a minimal size product. Residues S33, S34, K35 and R95 are shown as licorice, with C, O, N and P atoms in cyan, red, blue and ochre, respectively; only polar H atoms (white) are displayed. The remainder of the protein is represented in gray cartoon form.

Computational modeling was used to gain insight on how α _2_-α _3_ loop phosphorylation enhances catalytic efficiency, and the specific requirement for a serine phosphomonoester. A homology model of *Ec*-RNase III^29^ was used (Fig. 4) that contained a cleaved dsRNA substrate. This structure probably corresponds to an intermediate in the product release pathway, immediately following phosphodiester hydrolysis^36^. To assess local conformational changes as a consequence of S33 or S34 phosphorylation, molecular dynamics (MD) simulations were performed on *Ec*-RNase III and its two monophosphorylated forms (pS33 or pS34). Figure 5a and Supplemental Figure S8 show that the S33 phosphomonoester is engaged in a bidentate salt bridge with the R95 guanidinium group, and also a more flexible (i.e. weaker) ionic interaction with the K35 side chain. In contrast, the S34 phosphomonoester interacts primarily with K35 (Fig. 5b), with a salt bridge to R95 only infrequently observed (Suppl. Fig. S8, and Suppl. Tables S3 and S4). Simulations of the diphosphorylated (pS33 and pS34) RNase III-product complex exhibit the same pS33-R95 interaction (Suppl. Fig. S9) seen in the monophosphorylated (pS33) complex (Fig. 5a), providing further support for a dominant effect of S33 phosphorylation on repositioning the R95 side chain. R95 is a feature of RNA-binding motif 4 (RBM4) (see Fig. 1) which interacts with the 2 bp distal box (db) that is present in RNase III substrates >11 bp in length^37^. The db (see Fig. 3a) is important for substrate reactivity, as its deletion or mutation causes a loss of binding affinity^38^. In this regard, the dsRNAs used in the simulations are 9 bp in length with a 2 nt, 3′-overhang at each end, and do not contain a complete db (see Suppl. Fig. S2). Thus, the modeling would not be expected to show a direct R95 side chain interaction with the RNA. Moreover, other crystal structures of *Aa*-RNase III bound to cleaved RNAs exhibit RBM4-db interactions that are dependent upon RNA structure and the specific type of product complex (Suppl. Table S7). Furthermore, these observations may relate to the variability of RBM4 sequence and length among RNase III orthologs^37^,^38^. Despite the limitations of the homology-modeled structure, the S33 phosphorylation-dependent repositioning of the R95 side chain suggests a mechanism for facilitated product release, in which the ionic interaction of the R95 side chain with the substrate distal box is replaced by a bidentate ionic interaction with the S33 phosphomonoester.

The modeling also explains why glutamic acid substitution at position 33 or 34 does not confer a rate enhancement. The predicted interaction of R95 with a glutamic acid at position 33 (Suppl. Fig. S11a) is significantly weaker than the interaction involving the S33 phosphomonoester (Suppl. Fig. S12 and Suppl. Tables S3 and S4). Similarly, the interaction of K35 with E34 (Suppl. Fig. S11b) is less stable than with pS34. Moreover, MD simulations of the S33E, S34E double mutant (Suppl. Fig. S11c), which formally provides the same double-negative charge as a single S33 or S34 phosphomonoester, indicate that an additional acidic residue at position 34 does not provide a stabilized interaction with R95 (see Suppl. Fig. S12 and Suppl. Tables S3 and S4). In contrast to the bidentate pS33-R95 side chain interaction, the observed salt bridge consists of a monodentate engagement of R95 with the E33 side chain, and no involvement of the E34 side chain. We also note that Glu-Arg and Glu-Lys interactions generally are ~1–2 kcal/mol weaker than pSer-Arg or pSer-Lys interactions^39^. Hence, we conclude that the phosphomonoester group offers optimal geometric and electronic features that allow stable bidentate salt bridge formation with the R95 guanidinium group, and that the resulting weakened interaction between RBM4 and the distal box facilitates product release.

In the proposed model, serine phosphorylation also could be expected to weaken the binding of substrate as well as product. If so, this could counteract any rate enhancement provided by facilitated product release. However, we find that phosphorylation has only a minor effect on the stability of the enzyme-substrate complex (see Fig. S4). While a definitive explanation of how phosphorylation selectively acts on product release is not yet available, it should be noted that the product complex formally differs from the substrate complex by the presence of a doubly-negatively-charged phosphomonoester. The phosphomonoester may provide additional electrostatic destabilization of the product, compared to the substrate complex, and in turn could confer a greater sensitivity of the RBM4-db interaction to serine phosphorylation.

### Bioinformatics analysis of the α_2_-α_3_ phospholoop

Alignment of RNase III sequences from bacteria that are known hosts for T7-related phages (Suppl. Fig. S16) reveals a conservation of the α _2_-α _3_ phospholoop and R95. However, the overall sequence identity of the RNase III orthologs (Suppl. Table S9) is >90% if *Stenotrophomonas* and *Vibrio* are excluded, so regions in addition to the α _2_-α _3_ loop also are highly conserved. In contrast, a broadened alignment of RNase III sequences, including those from bacteria for which there is no evidence for a T7-related phage, shows an α _2_-α _3_ loop of variable length and a lower conservation of S33 and S34 (Suppl. Fig. S17). Why would a bacterial cell have phosphorylation sites that benefit phage reproduction? It is possible that T7PK may recognize a pre-existing regulatory network involving a cell-encoded protein kinase. A GenBank database search using T7PK as query failed to identify a putative cellular ortholog. However, it is possible that strong selective pressure on the phage genome may have caused significant sequence divergence from an ancestral, host-derived kinase gene. Alternatively, convergent evolution could have produced a phage protein kinase with similar target specificity.

## Materials and Methods

Chemicals and reagents were molecular biology grade and were obtained from Sigma-Aldrich or Thermo Fisher Scientific. Standardized 1M MgCl_2_ was from Sigma-Aldrich. Ribonucleoside 5′-triphosphates were obtained from Roche Molecular Biochemicals. The radiolabeled nucleotides [α -^32^P]UTP (3000 Ci/mmol) and [γ –^32^P]ATP (3000 Ci/mmol) were purchased from GE Healthcare Life Sciences. Lambda phage protein phosphatase and T4 polynucleotide kinase were purchased from New England BioLabs. Dialysis membranes (Spectra–Por CE 3500, 10,000 MWCO) were purchased from Thermo Fisher Scientific. Ni^2+^-NTA affinity chromatography resin, biotinylated thrombin and streptavidin-agarose were purchased from EMD Millipore. Protein assay kits and SDS-PAGE protein standards (low MW range) were from Bio-Rad Laboratories. ICON^TM^ concentrators, agarose, and NuPAGE Precast Bis-Tris or Tricine gels (12% and 15%, respectively) were purchased from Thermo Fisher. Oligodeoxynucleotides used for mutagenesis and for *in vitro* RNA synthesis were provided by Thermo Fisher in fully deprotected form, and were purified by denaturing gel electrophoresis, then stored at − 80 °C in 10 mM Tris, 1 mM EDTA (TE) buffer (pH 8.0) until further use.

### RNA synthesis

Internally ^32^P-labeled R1.1 RNA was synthesized *in vitro* using T7 RNA polymerase (purified in-house as described^40^) and an oligodeoxynucleotide template essentially as described^41^, using [α -^32^P]UTP (100 Ci/mol) as radiolabel. The concentration of all four rNTPs was 1 mM, and 400 units of T7 RNA polymerase was used, in 0.1 ml reactions. RNA was purified by electrophoresis in a 15% polyacrylamide gel containing 7M urea in Tris/Borate/EDTA, and stored at − 20 °C in TE buffer (pH 7.0).

### Mutagenesis and protein purification

Site-directed mutagenesis was accomplished using QuikChange multisite-directed mutagenesis kits (Agilent Technologies) and a single mutagenic oligodeoxynucleotide (sequences available on request). *Ec*-RNase III and mutant versions were overproduced in H6-tagged form using *E. coli* BL21(DE3)*recA,rnc105* cells^41^ that carried the respective recombinant pET-15b plasmid, and purified by affinity chromatography essentially as described^41^. Briefly summarized, cells were grown with vigorous aeration to mid-log phase at 37 °C in LB media containing ampicillin. Approximately 4 hours following addition of IPTG (1 mM final concentration), cells were collected by centrifugation at 4 °C. The pellet was resuspended in column loading buffer, sonicated until lysis was complete, then centrifuged and the clarified supernatant applied to a Ni^2+^-NTA column. Following repeated column washes, the fractions containing the eluted protein were collected and dialysed against 1 M NaCl, 60 mM Tris-HCl (pH 7.9), 1 mM EDTA, 1 mM DTT for 12 hr at 4 °C (EDTA and DTT can be omitted). An equal volume of glycerol was added and the protein stored at − 20 °C. The H6-tag was removed as needed by treatment with biotinylated thrombin as described^24^.

### T7PK phosphorylation of protein *in vitro*

T7 protein kinase (T7PK) was purified in H6-tagged form and dephosphorylated as described^24^. A standard phosphorylation assay involved an initial incubation of equimolar amounts of RNase III (or RIIID) and dephosphorylated T7PK for 5 min at 30 °C in 2 mM NH_4_Cl, 30 mM Tris-HCl (pH 7.2), 1 mM DTT, 0.1 mM EDTA. Then, MgCl_2_ was added (15 mM final concentration), followed by [γ ^32^-P]ATP (12 Ci/mol; 1 mM final concentration). Following a 10 min at 30 °C, an additional aliquot of T7PK was added, at half the original amount. After a 5 min incubation excess EDTA (20 mM final concentration) was added. Aliquots were combined with SDS-PAGE loading buffer, heated at 100 °C for 3 min and analyzed by SDS-PAGE. Proteins were visualized by Coomassie Brilliant Blue R staining and ^32^P-radioactivity detected by phosphorimaging (Typhoon 9400 System). To prepare phosphorylated nonradioactive RNase III for use in RNA cleavage and binding assays, phosphorylation reactions were performed using RNase III with the H6-tag enzymatically removed by thrombin treatment^24^, dephosphorylated T7PK, and a 1 mM ATP concentration. The reaction mixture was loaded onto a Ni^+2^-NTA spin column (Qiagen) and centrifuged at 500 ×  g for 5 min. Phosphorylated RNase III free from H6-T7PK was recovered in the eluted volume, dialysed against storage buffer and stored at a concentration of ~0.5 mg/ml in 50% glycerol (v/v) at − 20 °C until further use.

### Substrate cleavage assay

Substrate cleavage assays were performed essentially as described^41^ using internally ^32^P-labeled R1.1 RNA (see above, and Fig. 3) and purified WT or mutant H6-tagged RNase III. Briefly, assays were conducted by incubating R1.1 RNA and RNase III (specific amounts indicated in the relevant Figure and Table legends) in 160 mM NaCl, 30 mM Tris-HCl (pH 8) for 1 min at 37 °C. Reactions were initiated by adding MgCl_2_ (10 mM final concentration, unless otherwise specified), followed by incubation at 37 °C for the specified time. Aliquots were removed and combined with gel loading dye containing excess EDTA, then analyzed by electrophoresis in 15% (w/v) polyacrylamide gels containing 7 M urea and TBE buffer^41^. RNA was visualized by phosphorimaging (Typhoon 9400 system) and quantified using ImageQuant software. Curve fitting for kinetic parameter determination used Kaleidagraph software (v.3.5) (see also Table 2 legend). Single-turnover cleavage assay conditions are described in the legend to Supplemental Figure S5.

### Computational modeling

A homology model of *Ec*-RNase III was built as described^29^, and was based on the crystal structure of *Aquifex aeolicus* (*Aa*) RNase III (PDB entry 2NUG)^36^. The *Aa*-RNase III structure corresponds to a complex of the enzyme with the minimal product of dsRNA processing^37^, consisting of a 9 bp dsRNA with a 2-nt, 3′-overhang at each end, and is proposed to represent an early step in the product release pathway^36^. The product-bound homology model of *Ec*-RNase III was simulated in different states: (i) wild-type homodimer (wt–wt′); (ii) wild-type with both subunits phosphorylated at Ser33 (pS33–pS33′); (iii) wild-type with both subunits phosphorylated at Ser34 (pS34–pS34′); (iv) wild-type with both subunits diphosphorylated at Ser33 and Ser34 (pS33, pS34–pS33′, pS34′); (v) the S33E mutant-mutant homodimer (S33E–S33′E); (vi) the S34E mutant-mutant homodimer (S34E–S34′E); and (vii) the S33E, S34E double mutant homodimer (S33E, S34E–S33′E, S34′E). For each of the simulated systems, two independent 50 ns molecular dynamics (MD) simulations were performed in order to refine the homology models and explore possible local conformational changes due to phosphorylation or mutation. In the monophosphorylated states (pS33 or pS34) phosphoserine was modeled in doubly unprotonated form (*i.e.* with a − 2 charge), since the first and second pKa values of the phosphomonoester group are 2.19 and 5.78, respectively (see ref. 42 and references therein). In the diphosphorylated state (pS33 and pS34), two possible protonation states were considered: doubly unprotonated (− 2 charge) pS33 together with singly unprotonated (−1 charge) pS34, or *vice versa*. As a control for the homology modeling protocol, and taking into account that *Aa*-RNase III is also phosphorylated *in vitro* by T7PK (Suppl. Fig. S6), MD simulations were also performed for *Aa*-RNase III in complex with a minimal product dsRNA (PDB entry 2NUG)^36^, which is the same structure used as a template in the homology modeling. Phosphorylation was only considered at *Aa*-S31, *i.e.* the residue equivalent to *Ec*-S33 (see Fig. 1). The non-phosphorylated and phosphorylated forms of the *Aa*-RNase III enzyme were simulated for 100 ns each. All the MD simulations were performed using the NAMD program^43^, with additional details available in the Supplementary Information (SI). The computational images were generated with the programs ESPript^44^ (Fig. 1) and VMD^45^ (Figs 4 and 5).

## Additional Information

**How to cite this article**: Gone, S. *et al.* Mechanism of Ribonuclease III Catalytic Regulation by Serine Phosphorylation. *Sci. Rep.* **6**, 25448; doi: 10.1038/srep25448 (2016).

## Supplementary Material

Supplementary Information

## Figures and Tables

**Table 1:** T7PK phosphorylation of *Ec*-RNase III and double alanine mutants.

Protein^a^	Incorporation^b^ (mol phosphate/mol RNase III)
WT	0.32 ± 0.01
S33A, S34A	0.00 ± 0.01
S195A, S198A	0.27 ± 0.01

^a^Purified RNase III (WT) and the two double mutants, S33A, S34A, and S195A, S198A were phosphorylated by T7PK in the presence of 1 mM [γ -^32^P]ATP, as described in Materials and methods.

^b^Phosphate incorporation was measured by liquid scintillation counting of excised gel bands (see also Materials and methods). Numbers reported are the average of three experiments (± SEM), and correspond to mol phosphate incorporated per mol RNase III (homodimer). The listed values had the background incorporation value (0.04 mol phosphate/mol RNase III) subtracted. The background was defined by the radioactivity present in the same position (same sized gel slice) in a lane containing a phosphorylation reaction that omitted RIIID.

**Table 2:** Effect of phosphorylation on the kinetic parameters of substrate cleavage by *Ec*-RNase III.

Enzyme	K_m_ (nM)	k_cat_ (min^−1^)	k_cat_/K_m_ (M^−1^min^−1^)	k_2_ (min^−1^)
RNase III (+ P)	163 ± 26	1.74 ± 0.21	1.07 × 10^7^	2.5 ± 0.4
RNase III (− P)	239 ± 37	0.34 ± 0.29	1.45 × 10^6^	2.7 ± 0.7

Kinetic parameters were determined using internally ^32^P-labeled R1.1 RNA as substrate (see Materials and methods). RNase III(+ P) refers to *Ec*-RNase III subjected to phosphorylation by T7PK and ATP, followed by purification (see Materials and methods). RNase III (−P) refers to a mock phosphorylation reaction where MgCl_2_ was omitted from an otherwise complete reaction. Experiments were performed in duplicate. The K_m_ and k_cat_ values were determined by nonlinear least-squares curve-fitting (Kaleidagraph v3.5) of the data points (Fig. 3d) to a Michaelis-Menten scheme. The k_cat_ value was calculated by dividing V_max_ by the enzyme concentration. The standard errors are provided. The k_2_ value is the exponential decay constant for R1.1 RNA cleavage under single-turnover conditions. To determine the k_2_ values, reactions were performed as described in Materials and methods and the exponential decay constant obtained by fitting the time-course reaction progress curve to the single exponential equation, y =  m_1_ +  m_2_ · e^−m3t^, where t is time (min) and m_3_ is the decay constant k_2_ (min^−1^). The k_2_ values are the average of two experiments, and the maximum error values are provided.
