# Supplementary material for: Mechanism of Ribonuclease III Catalytic Regulation by Serine Phosphorylation
Source: Sci Rep. 2016 May 6;6:25448. doi: 10.1038/srep25448 (PMC4858673; doi:10.1038/srep25448)
Supplement: Supplementary Information [file srep25448-s1.pdf]

Mechanism of Ribonuclease III Catalytic Regulation by Serine Phosphorylation

Supplementary Information

Swapna Gone[1,¶,@] Mercedes Alfonso-Prieto[2,#,@], Samridhdi Paudyal[3,§],

and Allen W. Nicholson[1,3,*]

[1]Department of Chemistry, [2]Institute for Computational Molecular Science, and

[3]Department of Biology

Temple University, Philadelphia PA, 19122, USA

* Corresponding Author

@ These coauthors contributed equally to the study

Present address:

¶ Bioo Scientific Corporation, Austin, TX, USA, 78744

Department of Inorganic and Organic Chemistry, University of Barcelona, E-08028

Barcelona, Spain

§ Department of Pediatrics, Section of Hematology-Oncology, Baylor College of Medicine,

Houston, TX, USA, 77025

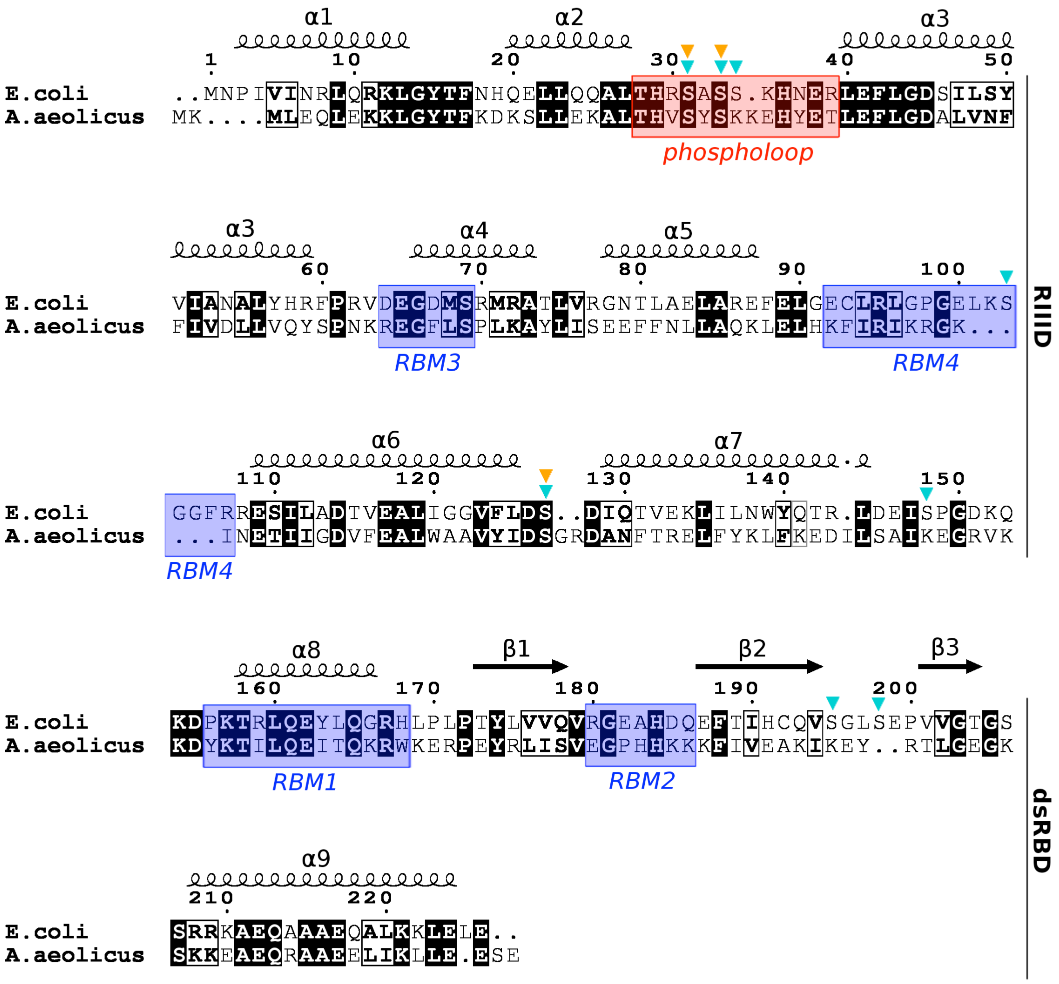

Figure S1. Alignment of the *Escherichia coli* (*Ec*) and *Aquifex aeolicus* (*Aa*) RNase III full-length polypeptide sequences, visualized with ESPript[1]. The residue numbering of *Ec*-RNase III and its secondary structure (predicted using PSIPRED[2]) are shown above the aligned sequences. Conserved residues are highlighted in black, and similar residues are boxed. The α_2-α_3 loop containing the phosphorylation target sites (phospholoop) is highlighted in red, and the RNA-binding motifs (RBM)[3] are in blue. The serine residues predicted to be at the protein surface (and thus possible targets for phosphorylation) are indicated by triangles above the aligned sequences. All surface serines are indicated for *Ec*-RNase III (cyan triangles), whereas for *Aa*-RNase III only the conserved serines are marked (orange).

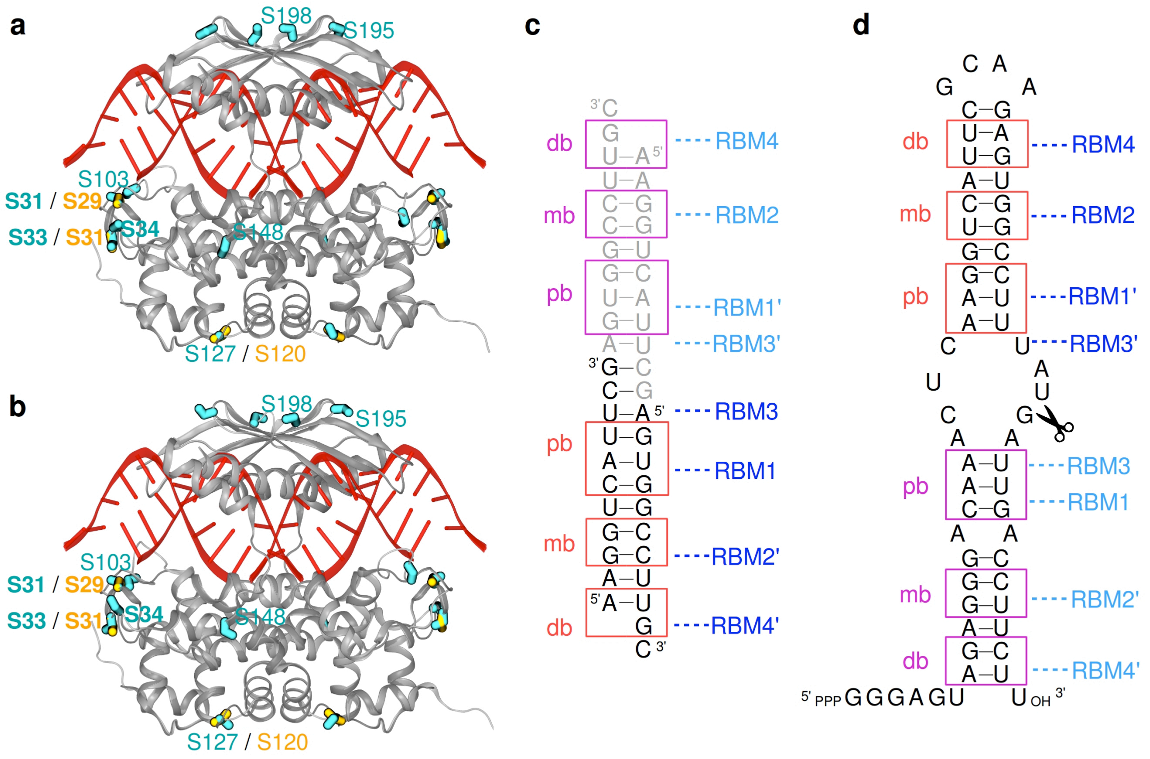

Figure S2. Structural models of *Ec*-RNase III used in the molecular dynamics simulations. **(a), (b)** Homology models of *Ec*-RNase III in a presumptive post-catalytic complex (replicas 1 and 2, respectively), built using as template the *Aa*-RNase III crystal structure with cleaved dsRNA bound to the catalytic valley (PDB entry 2NUG)[4]. The *Ec*-RNase III protein models are shown in light gray cartoon form, whereas the *Aa*-RNase III template protein is in dark gray. The cleaved dsRNA is displayed in red cartoon form. The serine residues on the protein surfaces are shown in ball-and-stick form; all serines (Ser, coloured in cyan) are displayed for *Ec*-RNase III, whereas for *Aa*-RNase III only conserved serines (coloured in orange) are shown. For better clarity, only one of the two subunits of the homodimer is labeled. **(c)** Sequence and secondary structure of the cleaved dsRNA (RNA 9, according to reference [4]) shown in panels (a) and (b). Two molecules are bound to *Ec*-RNase III in the model of the product complex, indicated in black and gray. The proximal, middle and distal boxes (pb, mb and db, respectively) are marked with red and

magenta boxes, depending on the dsRNA molecule, and the interactions with the RNA binding motifs (RBM1-4) in the RNase III polypeptide are indicated in blue and cyan, respectively; the prime superscript indicates the second subunit of the RNase III homodimer. **(d)** Sequence of the R1.1 RNA used in the experiments, showing the complete distal box that RNA 9 in panel (c) is missing. The site of cleavage under the experimental conditions employed in this study is indicated. The same colouring code as in panel (c) is used for the RNA boxes (pb, mb and db) and the RNase III motifs (RBM 1-4).

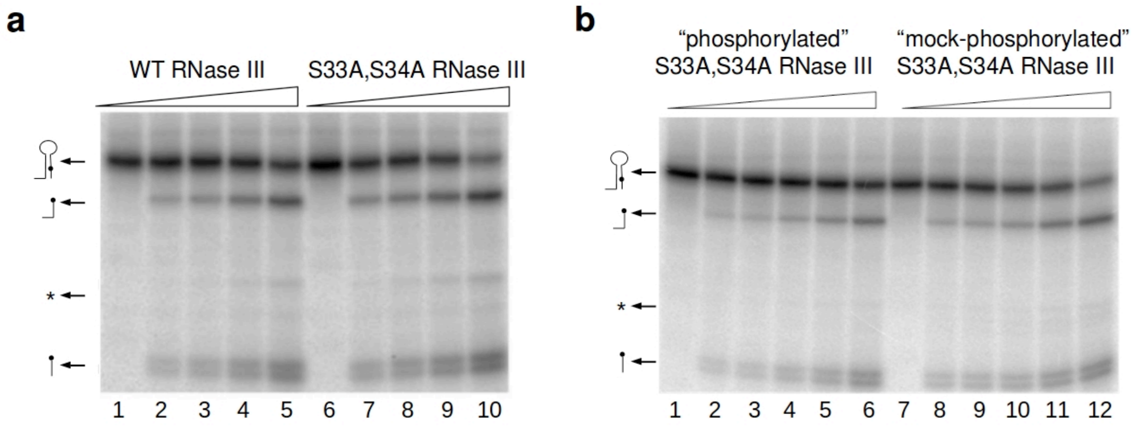

Figure S3. Time course cleavage assays for the S33A, S34A double mutant. **(a)** The double mutation itself does not alter RNase III activity. The cleavage assays used internally ^{32}P-labelled R1.1 RNA (100 nM, 10,000 dpm) in buffer consisting of 150 mM NaCl and 30 mM Tris-HCl, pH 8.0). Reactions were initiated by adding 10 mM $MgCl_2$, followed by incubation at 37°C. Lanes 2–5 and lanes 7–10 represent reaction times of 30 sec, 1 min, 2 min, and 5 min for wild-type RNase III (20 nM) and S33A,S34A RNase III (20 nM), respectively. Lanes 1 and 6 represent control reactions in which RNA and enzyme were incubated for 1 min in reaction buffer that lacked $MgCl_2$. Cleavage products are indicated by the arrows. **(b)** The phosphorylation-resistant double mutant is unaffected by treatment with T7PK (Fig. 2b). RNase III [S33A,S34A] was incubated with dT7PK, with or

without ATP ("phosphorylated" and "mock-phosphorylated" reactions, respectively). The pretreated RNase III [S33A,S34A] (20 nM) was then examined in a substrate cleavage assay, using the same conditions as in panel (a). Lanes 2–5 and lanes 7–10 represent reaction times of 1, 2.5, 5, and 10 min, respectively. Lanes 1 and 6 represent control lanes in which RNA and enzyme were incubated for 1 min in the absence of $MgCl_2$ (conditions in which RNase III is not catalytically active). Reactions were stopped by adding an equal volume of gel electrophoresis sample buffer, and were electrophoresed in a 15% polyacrylamide gel containing 7M urea. Reactions were visualized by phosphorimaging, and quantitated using ImageQuant software. The obtained cleavage rates are 0.13±0.11 and 0.18±0.09 pmol/min for "phosphorylated" and "mock-phosphorylated" RNase III [S33A,S34A], respectively.

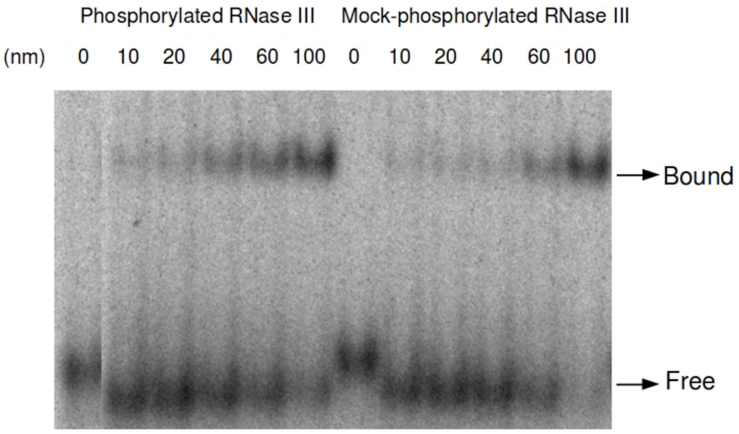

Figure S4. Gel electrophoretic mobility shift analysis of the effect of phosphorylation on the affinity of RNase III for R1.1 RNA, under noncatalytic conditions. The assay was performed as described in reference [5], using a buffer in which $MgCl_2$ was replaced by $CaCl_2$ (5 mM). R1.1 RNA was 5'-^{32}P-labeled, and 10^4 dpm (~50 pM concentration) was used in each reaction. The concentration of the protein (phosphorylated or mock-phosphorylated RNase III) is given on the top of each lane. The positions of free RNA and

the complex (bound) are indicated. Quantitation of the binding isotherms indicates similar substrate-binding behavior within experimental error; the calculated K'_D values are 67 and 79 nM for phosphorylated and nonphosphorylated RNase III, respectively.

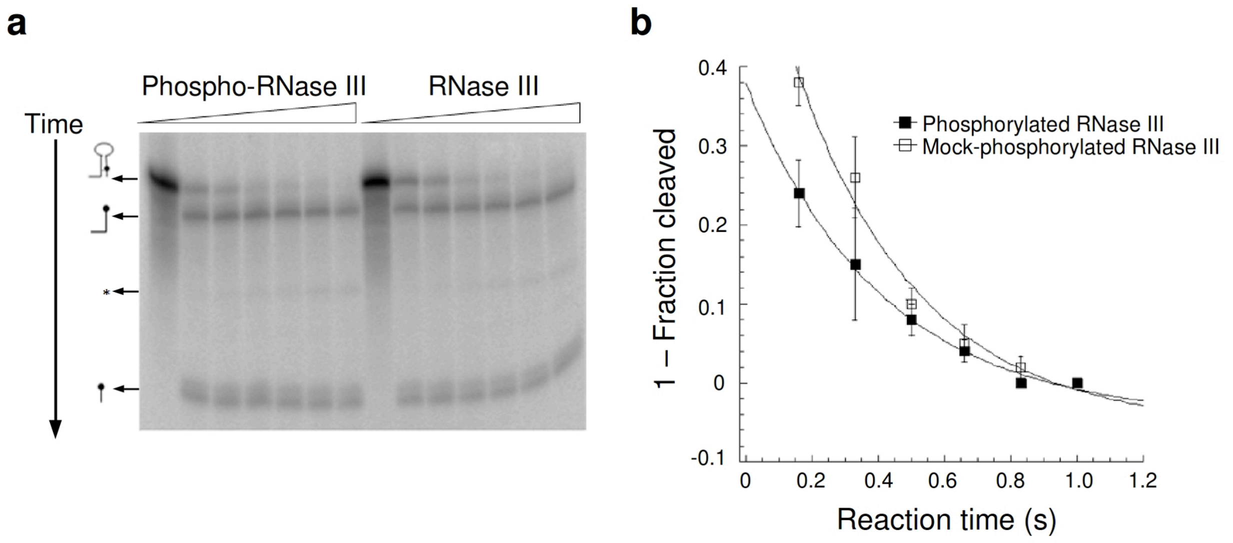

Figure S5. Phosphorylation of RNase III does not alter the rate of the hydrolytic step under single turnover conditions. In these assays, the ^{32}P-labeled R1.1 RNA concentration was 100 nM and the RNase III homodimer concentration was 120 nM. Reactions were set up and initiated as described in Materials and methods. Aliquots were removed at 10 sec intervals, up to 1 min, stopped with excess EDTA, and were analyzed by gel electrophoresis and phosphorimaging (see also Materials and methods). Decay constants (k_2) were determined as described in the Table 2 legend.

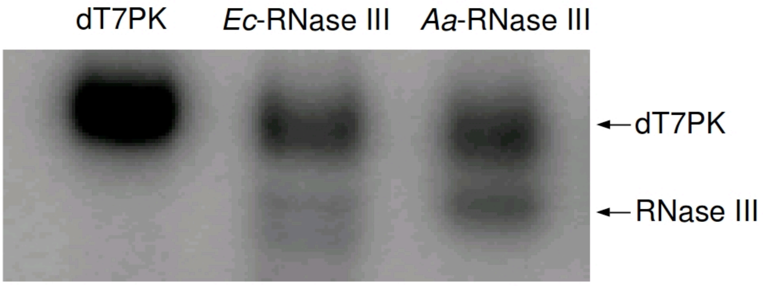

Figure S6. Phosphorylation of Aa-RNase III by T7PK. Reactions were performed and analyzed as described in Materials and methods, and in the legend to Figure 2. dT7PK refers to dephosphorylated T7PK. Lane 1 is a control reaction without added RNase III and shows only self-phosphorylated T7PK. Lanes 2 and 3 display phosphorylation reactions involving *Ec*-RNase III and *Aa*-RNase III, respectively.

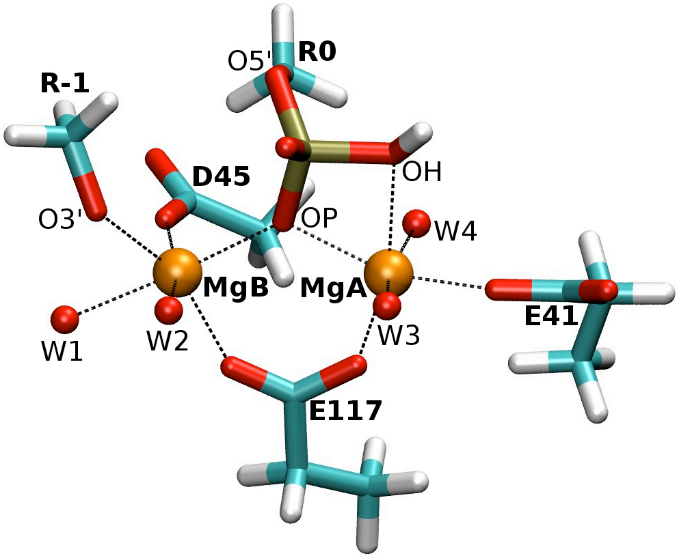

Figure S7. A model of the metal-containing catalytic site of RNase III used in the DFT-BLYP calculations. Residue numbering corresponds to *E. coli* RNase III. The hydrogen

atoms of the water molecules coordinated to MgB (W1 and W2) and to MgA (W3 and W4) are omitted to improve clarity.

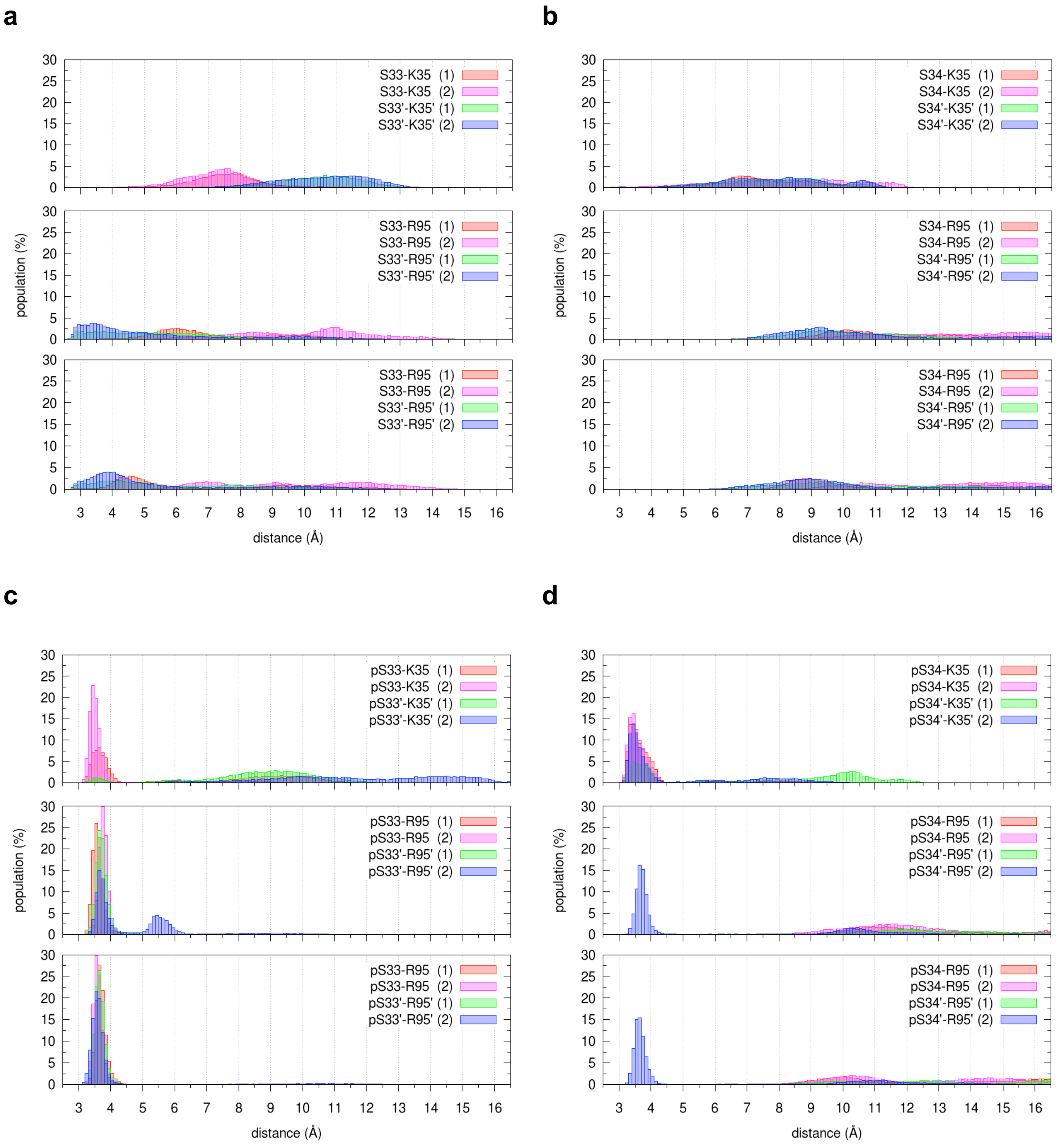

Figure S8. Interactions of S33 (*left*) and S34 (*right*) with K35 and R95 in the wild type *Ec*-RNase III homodimer. **(a)** S33 and **(b)** S34 in the nonphosphorylated state (wt–wt'); **(c)** pS33 in the monophosphorylated state (pS33–pS33'); **(d)** pS34 in the

monophosphorylated state (pS34–pS34'). In each panel, the top graph shows the distance from the amino nitrogen atom of K35 (N_{ζ}), and the center and bottom graphs the distance from the guanidinium nitrogen atoms of R95 (N_{H1} and N_{H2}, respectively). In the inset legends, (1) and (2) indicate two independent simulations started from the two different replicas obtained by homology modeling (see Fig. S2).

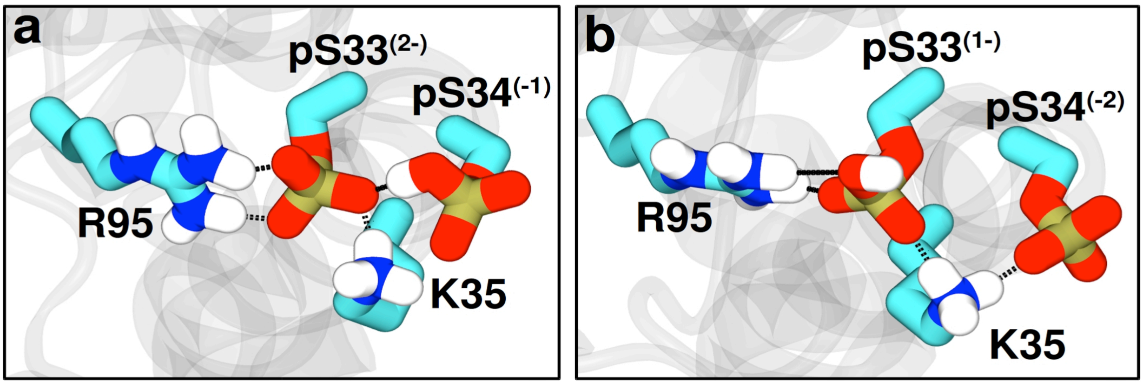

Figure S9. Representative interactions of pS33 and pS34 with nearby residues, as seen in the diphosphorylated form (pS33,pS34–pS33',pS34') of *Ec*-RNase III. **(a)** pS33 and **(b)** pS34 in the diphosphorylated state with a (2–) pS33 and a (1–) pS34; **(c)** pS33 and **(b)** pS34 in the diphosphorylated state with a (1–) pS33 and a (2–) pS34. Residues pS33, pS34, K35 and R95 are shown as licorice with C, O, N and P atoms in cyan, red, blue and ochre, respectively; only polar H atoms (in white) are displayed. The rest of the protein is represented as gray cartoon.

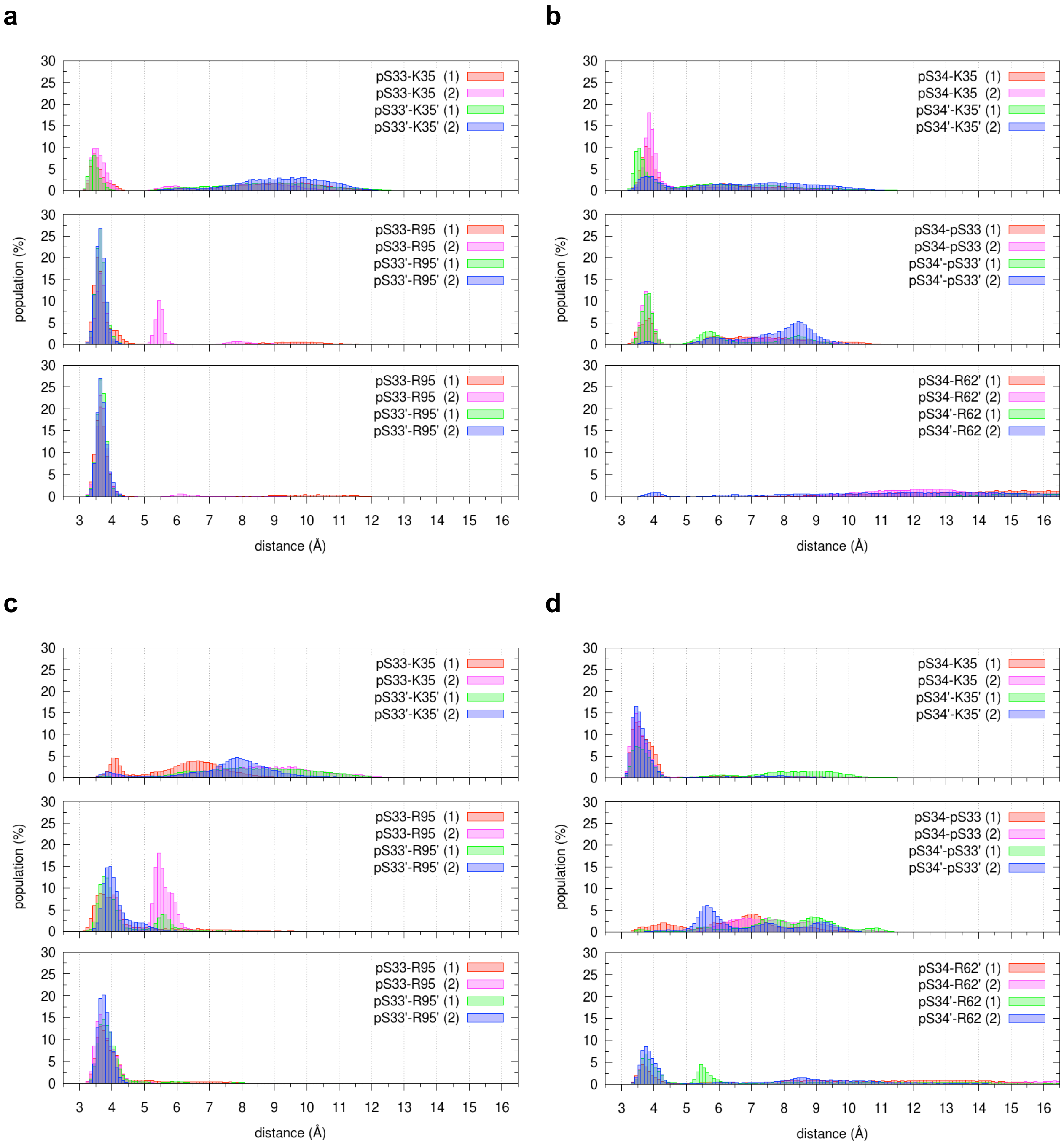

Figure S10. Interactions of pS33 (*left*) and pS34 (*right*) in the diphosphorylated state (pS33,pS34–pS33',pS34'). **(a)** pS33 and **(b)** pS34 in the diphosphorylated state with a (2−)

pS33 and a (1−) pS34; **(c)** pS33 and **(b)** pS34 in the diphosphorylated state with a (1−) pS33 and a (2−) pS34. In the (a) and (c) panels, the top graph shows the distance between pS33 (P) and K35 (N_ζ), and the center and bottom graphs the distance between pS33 (P) and R95 (N_{H1} and N_{H2}, respectively). In the (b) and (d) panels, the top graph shows the distance between pS34 (P) and K35 (N_ζ), the center graph the (P-O) distance between pS33 and pS34, and the bottom graph the distance between pS34 (P) and R62 (the closest guanidinium N atom, either N_{H1} or N_{H2}) In the inset legends, (1) and (2) indicate two independent simulations, started from the two different replicas (see Fig. S2) obtained by homology modelling.

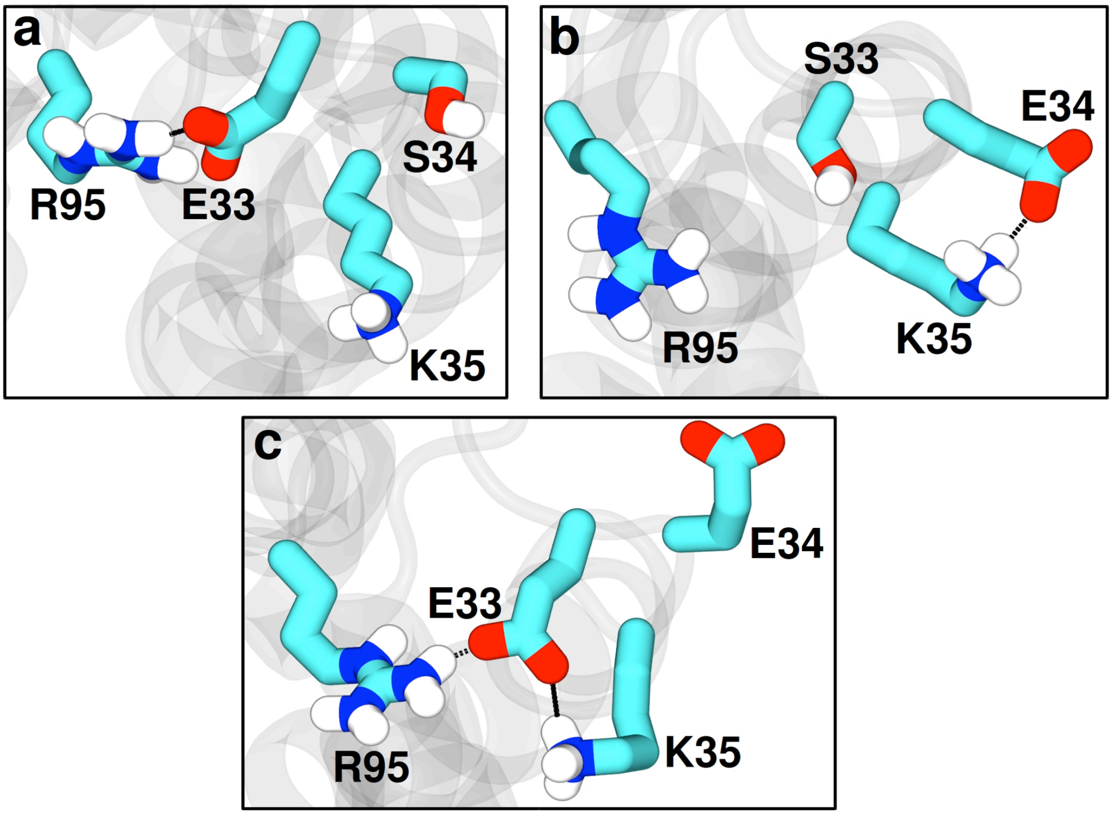

Figure S11. Representative interactions of residues 33 and 34 with nearby amino acids in the phosphomimetic *Ec*-RNase III mutants. **(a)** S33E mutant; **(b)** S34E mutant; **(c)**

S33E,S34E double mutant . Residues S33/E33, S34/E34, K35, and R95 are shown as licorice with C, O and N atoms in cyan, red and blue, respectively; only polar H atoms (in white) are displayed. The rest of the protein is represented as gray cartoon.

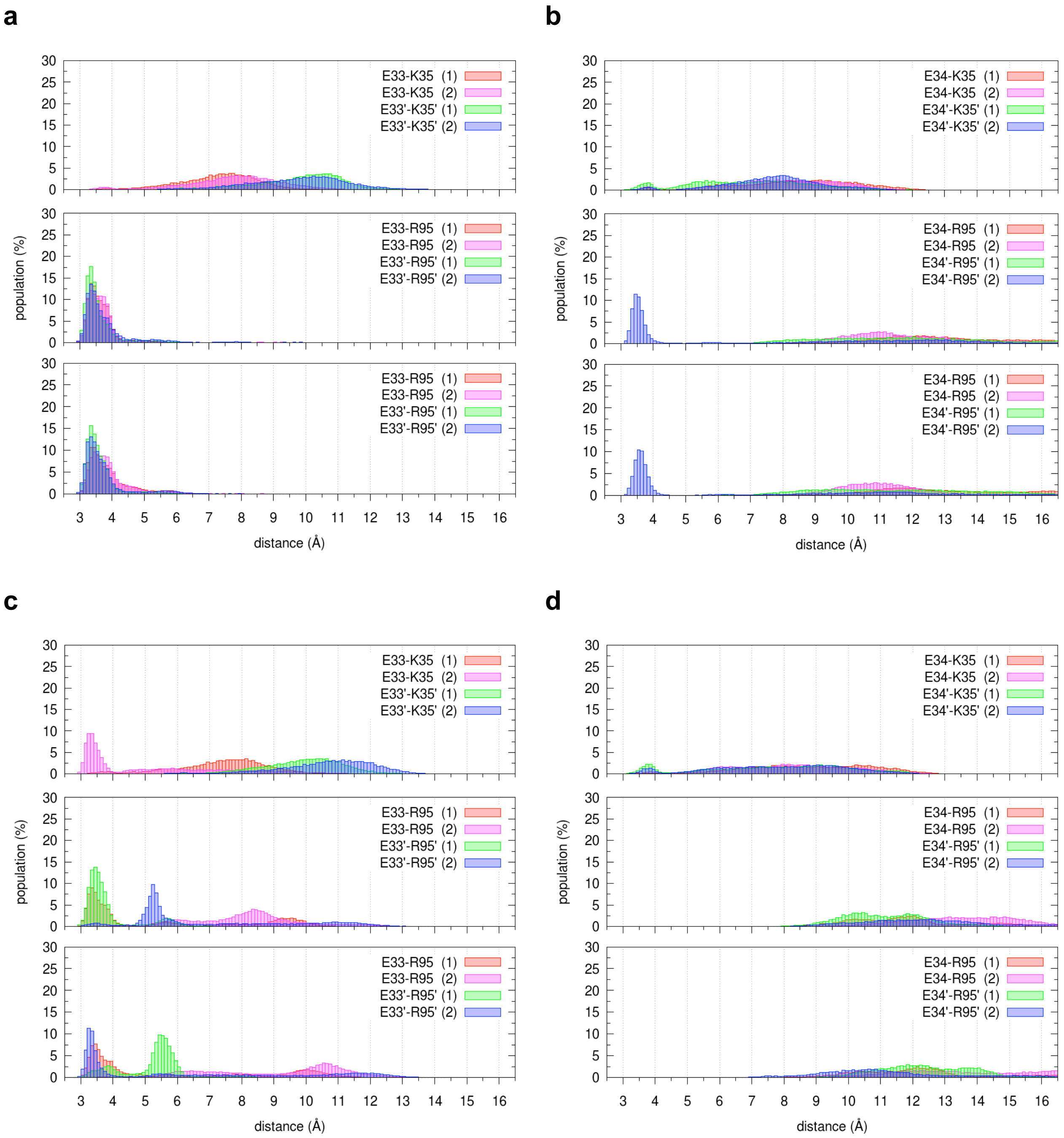

Figure S12. Interactions of E33 (*left*) and E34 (*right*) with K35 and R95 in the *Ec*-RNase III phosphomimetic mutants. **(a)** E33 in the S33E mutant (S33E–S33E'); **(b)** E34 in the S34E mutant (S34E–S34E'); **(c)** E33 and **(d)** E34 in the S33E,S34E double mutant (S33E,S34E–S33E',S34'E). In each panel, the top graph shows the distance from the amino nitrogen atom of K35 (N_ζ), and the center and bottom graphs the distance from the guanidinium nitrogen atoms of R95 (N_{H1} and N_{H2}, respectively). In the inset legends, (1) and (2) indicate the two independent simulations started from the two different replicas (see Fig. S2).

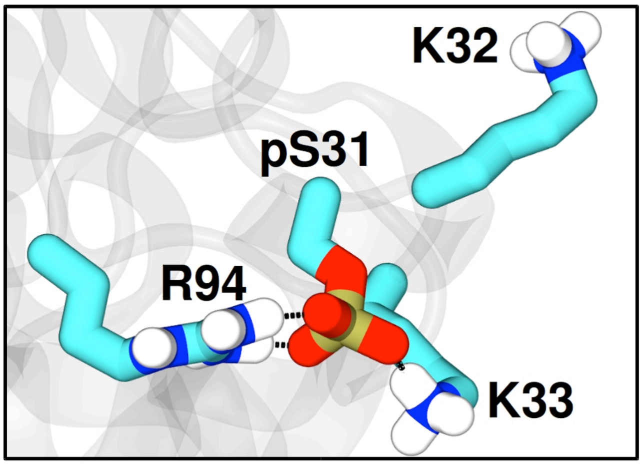

Figure S13. Representative interactions of pS31 with nearby residues in the phosphorylated form of *Aa*-RNase III. Residues pS31, K32, K33, and R94 are shown as licorice with C, O and N atoms in cyan, red and blue, respectively; only polar H atoms (in white) are displayed. The rest of the protein is represented as gray cartoon.

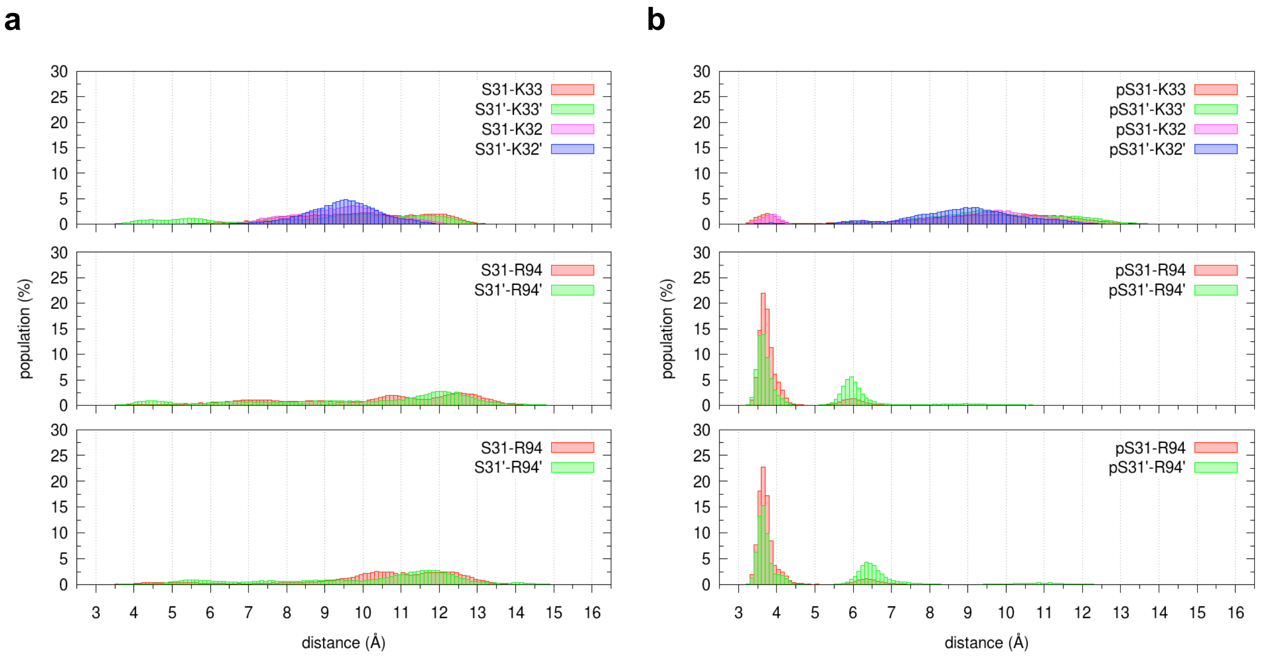

Figure S14. Interactions of S31 in nonphosphorylated and phosphorylated form with K33, K32, and R94 in the wild-type *Aa*-RNase III homodimer. **(a)** S31 in the nonphosphorylated state (wt–wt'); **(b)** pS31 in the phosphorylated state (pS31–pS31'). In each panel, the top graph shows the distance with the amino nitrogen atom of K33 and K32 ($N_ζ$) and the center and bottom graphs the distance with the guanidinium nitrogen atoms of R94 (N_{H1} and N_{H2}, respectively).

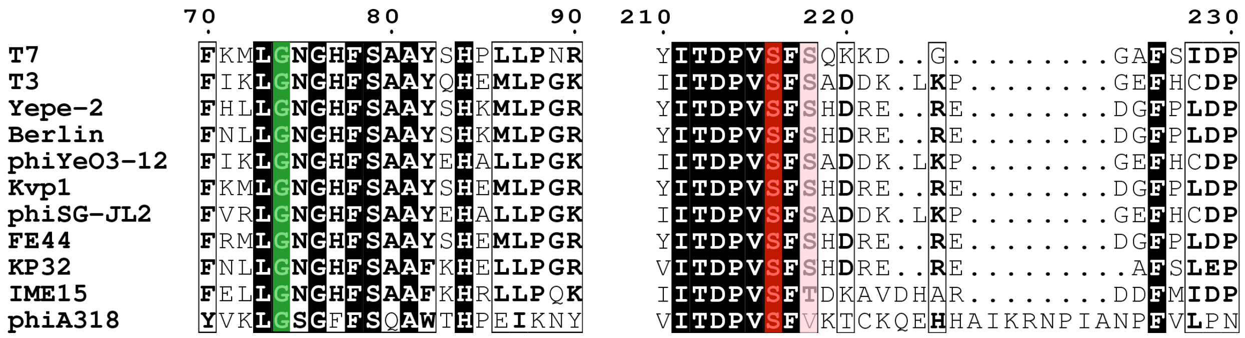

Figure S15. Alignment of partial sequences of predicted 0.7 protein orthologs in T7-like viruses. The residue numbering of T7 is shown above the aligned sequences. Only the functionally important regions spanning residues 70–90 and 210–230 are shown. Conserved residues are highlighted in black, and similar residues are boxed. Residue G76 of T7PK, implicated in ATP binding[6], is highlighted in green. Self-phosphorylated S216 and S218[7] are highlighted in red and pink, respectively.

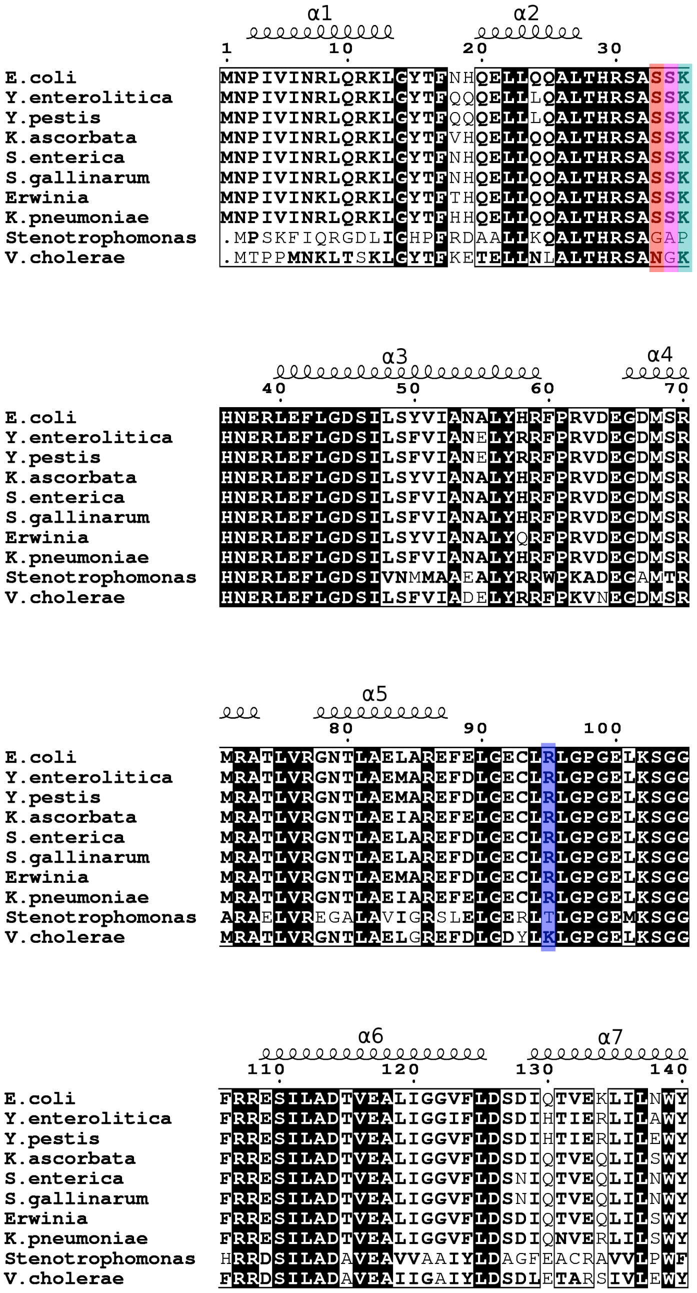

Figure S16. Sequence alignment of RNase III orthologs in bacteria infected by T7-like phages. Only the region corresponding to the RIIID is shown. The residue numbering of *Ec*-RNase III and its PSIPRED-predicted secondary structure[2] are included above the aligned sequences. Conserved residues are highlighted in black, and similar residues are boxed. The red- and pink-highlighted residues (S33 and S34 in *Ec*-RNase III) indicate the proposed phosphorylation sites. The cyan- and blue- highlighted residues (K35 and R95 in *Ec*-RNase III) indicate residues predicted to interact with phosphoserine.

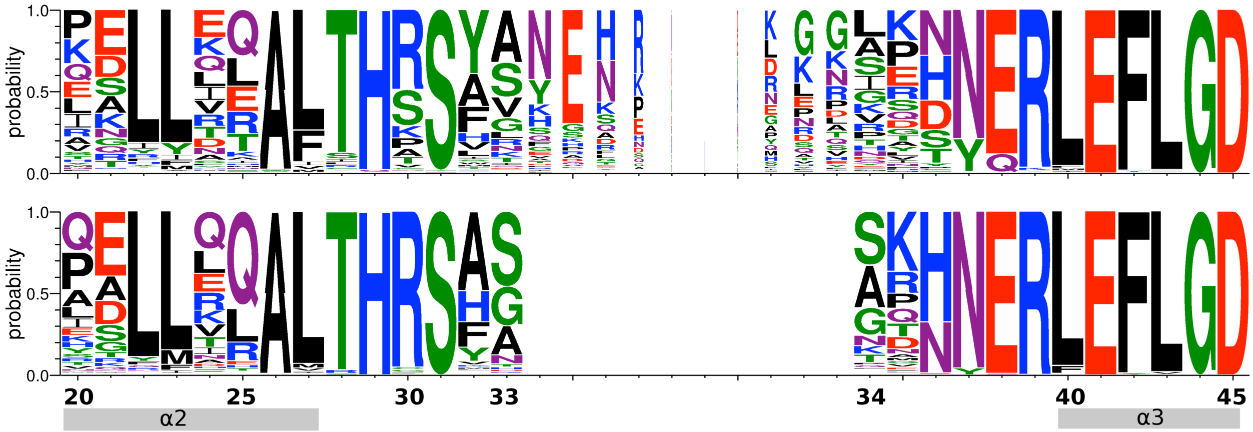

Figure S17. Sequence logo[8] of the RNase III phospholoop (residues 20-45; *Ec*-RNase III numbering), generated with WebLogo[9]. Amino acids A, V, I, L, M, W, F and P are in black; T, S, Y, C and G in green; R, K and H in blue; D and E in red; and N and Q in purple. The letter height is proportional to the probability of finding the corresponding amino acid at that particular position. *Top*, full sequence logo of the complete set of 1,138 RNase III sequences; the segment connecting helices α2 and α3 has a variable length. *Bottom*, biased sequence logo of a subset of 314 RNase III sequences with a α2–α3 loop of the same length as in *Ec*-RNase III.

Table S1. Main distances describing the metal-containing active site of RNase III. The calculated (DFT-BLYP) distances are compared with the experimental (X-ray) distances (as in PDB entry 2NUG[4]; the two values correspond to each of the catalytic sites of the *A. aeolicus* RNase III homodimer). Residue numbering corresponds to *E. coli* RNase III.

distance (Å)	X-ray	DFT-BLYP	distance (Å)	X-ray	DFT-BLYP
MgB-W1	2.08 / 2.04	2.26	MgA-W3	2.03/2.12	2.19
MgB-W2	2.32 / 2.21	2.26	MgA-W4	2.29/2.13	2.19
MgB-O3'(R-1)	2.15 / 2.07	2.13	MgA-OH(R-1)	2.23/2.10	2.30
MgB-OP(R0)	2.15 / 2.17	2.19	MgB-OP(R0)	2.29/2.19	2.14
MgB-D45	2.02 / 2.05	2.02	MgA-E41	2.03/1.97	1.99
MgB-E117	2.07 / 2.13	2.08	MgA-E117	2.18/2.21	1.98
MgB-MgA	3.91 / 3.99	3.83			

Table S2. Bader-corrected charges used for the metal-containing active site of RNase III. Residue numbering corresponds to *E. coli* RNase III.

residue	atom	charge (e^-)	residue	Atom	charge (e^-)
MgB	Mg	1.861	MgA	Mg	1.848
R-1	C3'	0.220	R0	C5'	0.074
	H3'	0.080		O5'	-0.481
	O3'	-1.199		P	1.166
D45	C_β	-0.022		O_P	-0.758
	H_β	-0.004		O_H	-0.560
	C_γ	0.808		H_O	0.288
	O_δ	-0.793	E41	C_γ	0.024
E117	C_γ	0.016		H_γ	-0.032
	H_γ	-0.040		C_δ	0.816
	C_δ	0.808		O_ε	-0.808

	O_ε	-0.817

Table S3. Hydrogen bond / salt bridge percentage occurrence in the *Ec*-RNase III simulated systems. An interaction is considered to be present when the corresponding distance is shorter than 3.5 Å (for the O_γ–N_ζ, O_γ–N_{H1} and O_γ–N_{NH2} distances) or 4.0 Å (for the P–N_ζ, P–N_{H1}, P–N_{H2} and C_δ–N_ζ, C_δ–N_{H1} and C_δ–N_{NH2} distances). The colour-coding is the same as in Figs. S8, S10 and S12, and the average occurrences are shown in black.

		occurrence (%)		
**state**	**Residue**	**K35 (N_ζ)**	**R95 (N_{H1})**	**R95 (N_{H2})**
**wt–wt'**	S33 / S34 (1)	0.0 / 0.4	0.3 / 0.0	1.6 / 0.0
	S33 / S34 (2)	0.0 / 1.0	0.0 / 0.0	0.0 / 0.0
	S33' / S34' (1)	0.0 / 0.4	12.9 / 0.0	9.1 / 0.0
	S33' / S34' (2)	0.0 / 0.4	20.3 / 0.0	12.9 / 0.0
	**(average)**	**0.0 / 0.6**	**8.4 / 0.0**	**5.9 / 0.0**
**pS33–pS33'**	pS33 (1)	37.8	81.6	79.3
	pS33 (2)	71.5	75.3	79.1
	pS33' (1)	4.9	78.2	80.8
	pS33' (2)	0.0	42.9	70.6
	**(average)**	**28.6**	**69.5**	**77.5**
**pS34–pS34'**	pS34 (1)	62.8	0.0	0.0
	pS34 (2)	65.6	0.0	0.0
	pS34' (1)	25.6	0.0	0.0
	pS34' (2)	50.9	48.8	49.3
	**(average)**	**51.2**	**12.2**	**12.3**
**$pS33^{(2-)}$,$pS34^{(1-)}$–$pS33'^{(2-)}$,$pS34'^{(1-)}$**	pS33 (1)	28.2	59.9	65.4
	pS33 (2)	40.3	47.8	69.0
	pS33' (1)	23.7	77.4	77.4
	pS33' (2)	0.2	78.0	75.6
	**(average)**	**23.1**	**65.8**	**71.9**
	pS34 (1)	34.2	0.0	0.0
	pS34 (2)	42.0	0.0	0.0
	pS34' (1)	32.5	0.0	0.0
	pS34' (2)	13.6	0.0	0.0

	(average)	30.6	0.0	0.0

Table S3. (*continued*) Hydrogen bond / salt bridge percentage occurrence in the *Ec*-RNase III simulated systems.

		occurrence (%)		
**state**	**Residue**	**K35 (N_ζ)**	**R95 (N_{H1})**	**R95 (N_{H2})**
**pS33$^{(1-)}$,pS34$^{(2-)}$–pS33'$^{(1-)}$,pS34'$^{(2-)}$**	pS33 (1)	5.0	41.2	49.2
	pS33 (2)	0.3	0.0	59.0
	pS33' (1)	1.1	44.4	54.0
	pS33' (2)	3.6	39.0	68.5
	**(average)**	**2.5**	**31.2**	**57.7**
	pS34 (1)	61.8	0.0	0.0
	pS34 (2)	63.1	0.0	0.2
	pS34' (1)	35.5	0.0	0.0
	pS34' (2)	66.5	0.0	0.0
	**(average)**	**56.7**	**0.0**	**0.0**
**S33E–S33'E**	E33 (1)	0.9	70.6	54.1
	E33 (2)	2.1	70.1	55.5
	E33' (1)	0.2	70.6	70.7
	E33' (2)	0.0	62.7	65.2
	**(average)**	**0.8**	**68.5**	**61.4**
**S34E–S34'E**	E34 (1)	1.9	0	0
	E34 (2)	6.5	0	0
	E34 (1)	2.7	0	0
	E34' (2)	2.4	39.5	39.2
	**(average)**	**3.4**	**9.9**	**9.8**
**S33E,S34E–S33'E,S34'E**	E33 / E34 (1)	2.3 / 0.8	42.7 / 0.0	36.3 / 0.0
	E33 / E34 (2)	39.9 / 1.9	0.2 / 0.0	0.4 / 0.0
	E33' / E34' (1)	0.0 / 8.5	64.8 / 0.0	13.3 / 0.0
	E33' / E34' (2)	0.0 / 4.8	4.4 / 0.0	36.1 / 0.0
	**(average)**	**10.6 / 4.0**	**28.0 / 0.0**	**21.5 / 0.0**

Table S4. Percent occurrences of hydrogen bonds / salt bridges in the *Ec*-RNase III simulated systems. The cutoff distances for the interactions are the same as in Table S3. The colour-coding is the same as in Figs. S8, S10 and S12, and the average occurrences are in black. “Mono” stands for monodentate interaction (residue 33 or 34 is interacting with only one of the guanidinium nitrogen atoms of Arg95), while “bi” denotes a bidentate interaction (simultaneous interaction with two guanidinium nitrogen atoms).

		occurrence (%)				
**state**	**residue**	**only K35**	**only R95 (mono)**	**only R95 (bi)**	**K35 + R95 (mono)**	**K35 + R95 (bi)**
**wt–wt'**	S33 / S34 (1)	0.0 / 0.4	1.4 / 0.0	0.2 / 0.0	0.0 / 0.0	0.0 / 0.0
	S33 / S34 (2)	0.0 / 1.0	0.0 / 0.0	0.0 / 0.0	0.0 / 0.0	0.0 / 0.0
	S33' / S34' (1)	0.0 / 0.4	12.7 / 0.0	4.7 / 0.0	0.0 / 0.0	0.0 / 0.0
	S33' / S34' (2)	0.0 / 0.4	18.8 / 0.0	7.2 / 0.0	0.0 / 0.0	0.0 / 0.0
	**(average)**	**0.0 / 0.6**	**8.2 / 0.0**	**3.0 / 0.0**	**0.0 / 0.0**	**0.0 / 0.0**
**pS33–pS33'**	pS33 (1)	0.1	3.9	41.7	1.7	36.0
	pS33 (2)	0.1	0.7	7.9	4.7	66.7
	pS33' (1)	0.0	7.3	71.2	0.5	4.4
	pS33' (2)	0.0	28.8	42.3	0.0	0.0
	**(average)**	**0.1**	**10.2**	**40.8**	**1.7**	**26.8**
**pS34–pS34'**	pS34 (1)	62.8	0.0	0.0	0.0	0.0
	pS34 (2)	65.6	0.0	0.0	0.0	0.0
	pS34' (1)	25.6	0.0	0.0	0.0	0.0
	pS34' (2)	19.5	1.9	18.2	3.1	28.4
	**(average)**	**43.4**	**0.5**	**4.6**	**0.8**	**7.1**
**pS33$^{(2-)}$,pS34$^{(1-)}$–pS33'$^{(2-)}$,pS34'$^{(1-)}$**	pS33 (1)	8.0	8.9	38.6	1.1	19.0
	pS33 (2)	0.0	3.7	52.6	1.3	22.3
	pS33' (1)	0.1	24.3	7.8	3.4	36.8
	pS33' (2)	0.0	5.7	73.7	0.0	0.2
	**(average)**	**2.0**	**10.7**	**43.2**	**1.5**	**19.6**
	pS34 (1)	34.2	0.0	0.0	0.0	0.0
	pS34 (2)	32.5	0.0	0.0	0.0	0.0
	pS34' (1)	42.0	0.0	0.0	0.0	0.0
	pS34' (2)	13.6	0.0	0.0	0.0	0.0

	(average)	30.6	0.0	0.0	0.0	0.0

Table S4. (*continued*) Percent occurrences of hydrogen bonds / salt bridges in the *Ec*-RNase III simulated systems.

		occurrence (%)				
**state**	**residue**	**only K35**	**only R95 (mono)**	**only R95 (bi)**	**K35 + R95 (mono)**	**K35 + R95 (bi)**
**pS33$^{(1-)}$,pS34$^{(2-)}$–pS33'$^{(1-)}$,pS34'$^{(2-)}$**	pS33 (1)	2.2	23.1	31.6	1.6	1.2
	pS33 (2)	0.1	32.0	32.3	0.3	0.7
	pS33' (1)	0.0	58.8	0.0	0.2	0.4
	pS33' (2)	0.4	32.6	35.6	2.7	0.5
	**(average)**	**0.7**	**36.6**	**24.9**	**1.2**	**0.7**
	pS34 (1)	61.8	0.0	0.0	0.0	0.0
	pS34 (2)	35.5	0.0	0.0	0.0	0.0
	pS34' (1)	62.9	0.0	0.0	0.2	0.0
	pS34' (2)	66.5	0.0	0.0	0.0	0.0
	**(average)**	**56.7**	**0.0**	**0.0**	**0.0**	**0.0**
**S33E–S33'E**	E33 (1)	0.1	21.3	51.1	0.4	0.5
	E33 (2)	0.1	20.2	51.1	1.0	1.1
	E33' (1)	0.0	6.2	67.4	0.0	0.2
	E33' (2)	0.0	16.4	55.7	0.0	0.0
	**(average)**	**0.1**	**16.0**	**56.3**	**0.4**	**0.5**
**S34E–S34'E**	E34 (1)	1.9	0	0	0	0
	E34 (2)	6.5	0	0	0	0
	E34' (1)	2.7	0	0	0	0
	E34' (2)	2.4	2.7	38.0	0	0
	**(average)**	**3.4**	**0.7**	**9.5**	**0**	**0**
**S33E,S34E–S33'E,S34'E**	E33 / E34 (1)	1.2 / 0.8	14.4 / 0.0	31.5 / 0.0	0.5 / 0.0	0.6 / 0.0
	E33 / E34 (2)	39.8 / 1.9	0.5 / 0.0	0.1 / 0.0	0.0 / 0.0	0.0 / 0.0
	E33' / E34' (1)	0.0 / 8.5	61.2 / 0.0	8.4 / 0.0	0.0 / 0.0	0.0 / 0.0
	E33' / E34' (2)	0.0 / 4.8	40.3 / 0.0	0.1 / 0.0	0.0 / 0.0	0.0 / 0.0
	**(average)**	**10.3 / 4.0**	**29.1 / 0.0**	**10.0 / 0.0**	**0.1 / 0.0**	**0.2 / 0.0**

Table S5. Percent occurrences of hydrogen bonds / salt bridges for each of the two *Aa*-RNase III systems. An interaction is considered to be present when the corresponding distance is either less than 3.5 Å ($O_γ$–$N_ζ$, $O_γ$–N_{H1} and $O_γ$–N_{NH2} distances) or less than 4.0 Å (P–$N_ζ$, P–N_{H1} and P–N_{H2} distances). The colour coding is the same as in Fig. S14.

		occurrence (%)			
**state**	**residue**	**K33 ($N_ζ$)**	**K32 ($N_ζ$)**	**R94 (N_{H1})**	**R94 (N_{H2})**
**wt–wt'**	S31	0.0	0.0	0.1	0.1
	S31'	0.1	0.0	0.1	0.2
	**average**	**0.1**	**0.0**	**0.1**	**0.2**
**pS31–pS31'**	pS31	10.2	0.1	71.9	72.3
	pS31'	0.6	1.2	48.8	47.1
	**average**	**5.4**	**0.7**	**60.4**	**59.7**

Table S6. Percent occurrence of hydrogen bonds / salt bridges for each of the two *Aa*-RNase III simulated systems. The cutoff distances for the interactions are the same as in Table S5. The colour-coding is the same as in Fig. S14, and the average occurrences are in black. “Mono” stands for monodentate interaction (only one of the guanidinium nitrogen atoms of the Arg side chain is interacting with residue 31), whereas “bi” denotes a bidentate interaction (two guanidinium nitrogen atoms of the Arg side chain forming interactions).

		occurrence (%)					
**state**	**residue**	**only K33**	**only K32**	**only R94 (mono)**	**only R94 (bi)**	**K33 / K32 + R94 (mono)**	**K33 / K32 + R94 (bi)**
**wt–wt'**	S31	0.0	0.0	0.3	0.0	0.0 / 0.0	0.0 / 0.0
	S31'	0.1	0.1	0.3	0.0	0.0 / 0.0	0.0 / 0.0
	**average**	**0.1**	**0.1**	**0.3**	**0.0**	**0.0 / 0.0**	**0.0 / 0.0**
**pS31–pS31'**	pS31	0.1	4.6	8.2	64.2	0.9 / 0.6	9.3 / 3.2
	pS31'	0.6	0.8	5.2	45.8	0.0 / 0.0	0.0 / 0.0

	**average**	**0.4**	**2.7**	**6.7**	**55.0**	**0.5 / 0.3**	**4.7 / 1.6**

Table S7. Available crystal structures of (*Aa*-)RNase III in complex with cleaved RNA products, and residues that interact with the distal box.

**PDB code**[a]	**complex**[b]	**dsRNA bound**[c]	**distal box interactions**[d]	
2NUG	product-0	2 x RNA 9	H27	K99, N101, T103
2NUF	product-2	2 x RNA 8	H27, K32	R97, N101, T103
4M2Z	product-2'	2 x RNA 10 + CMP	H27, K32	R97, N101, T103
4M30	product-2''	2 x RNA 12 + AMP	H27	R97, N101, T103

[a] See reference [4] for PDB codes 2NUG and 2 NUF, and reference [10] for 4M2Z.

[b] Product-0 (product complex immediately after phosphodiester cleavage) and product-1 (product complex in a subsequent step in the product release pathway) are the result of classic (type I) RNA cleavage events generating 2-nt 3' overhangs (RNA 7 → RNA 8 → RNA 9, see reference [4]). Afterwards, an additional (type II) RNA cleavage event can take place, yielding a 3-nt 3' overhang and a free nucleotide (see reference [10]). in particular, type II processing of RNA 8 (product-2) generates RNA 10 and a free CMP (here denoted as product-2'). Similarly, type II cleavage of RNA 6 (i.e. an oligo with the same sequence as RNA 8 except for the last bp and 2-nt 3' overhang) generates RNA 12 and a free AMP (here denoted as product-2'').

[c] RNA 9 = 9 bp with a 2 nt, 3'-overhang at each end (see Fig. S2); RNA 8 = 11 bp with a 4 nt stem loop on one end and a 2 nt 3' overhang on the other; RNA 10 = 10 bp with a 4nt stem loop on one end and a 3-nt 3' overhang; RNA 12 = same as RNA 10, except for the sequence of the 3-nt 3' overhang.

[d] H27 and K32 (*A. aeolicus* numbering) are located in the phospholoop; the equivalent residues in *E. coli* are H29 and K35, respectively. *Aa*-R97, K99, N101 and T103 belong to the RBM4; the equivalent residues are *Ec*-P98, E100, R108 and S110, respectively. The

protein-RNA interactions involve the phosphate-sugar backbone of the RNA distal box.

Table S8. Predicted 0.7 protein orthologs in T7-like viruses. The full-length 0.7 protein of T7 contains the T7PK domain as the N-terminal region (see [7] and references therein). The sequence identity calculated with respect to T7 0.7 protein is shown in the last column.

**Phage**	**Bacterial host**	**GenBank**	**UniProt**	**Identity**
T7	*Enterobacteria*	NP_041959	P00513	100%
T3	*Enterobacteria*	CAC86263	Q8W5U6	46.7%
Yepe-2	*Yersinia*	YP_002003314	B3VCH8	49.4%
Berlin	*Yersinia*	YP_918985	A0ZXJ8	50.0%
ΦYeO3-12	*Yersinia*	NP_052070	Q9T146	48.2%
Kvp1	*Kluyvera*	YP_002308385	B6Z9E5	54.1%
ΦSG-JL2	*Salmonella*	YP_001949749	B3FYH5	47.6%
FE44	*Erwinia*	YP_008766718	U5P049	53.4%
KP32	*Klebsiella*	YP_003347521	D1L2T6	53.5%
IME15	*Stenotrophomonas*	YP_006990206	K4NZU2	45.6%
ΦA318	*Vibrio*	YP_009110716	A0A067YBH9	32.5%

Table S9. Predicted RNase III orthologs in bacteria infected by T7-like viruses containing a 0.7 protein with predicted phosphotransferase activity. The sequence identity, calculated with respect to *E. coli* RNase III, is provided in the fourth column.

**Bacterial host**	**GenBank**	**UniProt**	**identity**	**Phage**
Escherichia coli	NP_417062	P0A7Y0	100%	T7, T3
Yersinia enterocolitica	YP_001005350	A1JKK3	91.2%	Yepe-2, Berlin, ΦYeO3-12
Yersinia pestis	NP_668620	Q8ZD72	91.6%	Yepe-2, Berlin, ΦYeO3-12
Kluyvera sp.	KFD08759	A0A085IKL3	97.8%	Kvp1
Salmonella enterica	YP_008248495	I0MTJ9	97.4%	ΦSG-JL2
Salmonella gallinarum	YP_002227478	B5RD49	97.4%	ΦSG-JL2
Erwinia sp.	YP_005816652	E3DBM2	92.0%	FE44
Klebsiella pneumoniae	YP_002237090	B5XNH0	96.9%	KP32
Stenotrophomonas sp.	WP_008267764	B8L6K6	47.1%	IME15
Vibrio cholerae	YP_002811133	C3LR04	69.0%	ΦA318

Protein sequence alignment

The sequence alignment of the ribonuclease III (RNase III) polypeptide sequences of *Escherichia coli* (*Ec*) and *Aquifex aeolicus* (*Aa*) is shown in Fig. S1 (full-length) and Fig. 1 (only the ribonuclease III domain, RIIID). For more details on the alignment protocol see reference [11].

The Hidden Markov Model-based sequence alignment presented here is slightly different from the structure-based sequence alignment provided by Ji and coworkers[10] in the region connecting helices α_2 and α_3. The differing lengths of the α_2–α_3 segment as well as its lack of structure renders a precise positioning of *Ec*-S34 and *Ec*-K35 unreliable.

Homology modeling

Homology models of *Ec*-RNase III were built based on the crystal structure of *Aa*-RNase III (PDB entry 2NUG)[4]. The *Aa*-RNase III structure corresponds to RNase III in a complex with two molecules of a cleaved dsRNA that has been proposed to be the minimal product of dsRNA processing[4], consisting of a 9 bp dsRNA with a 2-nt, 3'-overhang at each end. The minor conformation was chosen out of the two alternate conformations present in the 2NUG crystal[4], as it is proposed to represent the complex immediately after phosphodiester cleavage, corresponding to an initial step in product release. The sequence identity between *Ec*- and *Aa*-RNase III is 34% and the sequence similarity is 57%. Therefore, the quality of the resulting homology model is expected to be in the medium-to-high range[12], with ModEval[13] predicting a C_α RMSD of 2.60 Å for the model with respect to the native structure. Modeller (version 9.10)[14] was used for pairwise structure-sequence alignment and homology model building. 1,500 models were generated, then clustered using the g_cluster tool of the Gromacs package[15]. Using the Jarvis-Patrick algorithm[16] and applying a 0.5 Å cutoff, a nearest-neighbour list of 10 and a

shared-neighbor threshold of 3, two main clusters were obtained with populations of 76% and 23%. A representative structure of each of these clusters was selected and further refined by optimizing the conformation of the variable loops (residues 102-107 and 194-201), also using Modeller[17]. The resulting two models (denoted hereafter replicas 1 and 2; see Fig. S2) are practically identical, with a backbone RMSD of 0.73 Å.

Molecular dynamics (MD) simulations

The model of *Ec*-RNase III bound to product was subjected to MD simulations in different states: (i) wild-type, non-phosphorylated homodimer (wt–wt'); (ii) wild-type with both subunits phosphorylated on Ser33 (pS33–pS33'); (iii) wild-type with both subunits phosphorylated on Ser34 (pS34–pS34'); (iv) wild-type with both subunits diphosphorylated at Ser33 and Ser34 simultaneously (pS33,pS34–pS33',pS34'); (v) the S33E mutant-mutant homodimer (S33E–S33'E); (vi) the S34E mutant-mutant homodimer (S34E–S34'E); and (vii) the S33E,S34E double mutant homodimer (S33E,S34E–S33'E,S34'E). Each of the models was immersed in a box of water molecules with 150 mM NaCl, resulting in a system size of ~120,000 atoms. Two independent, 50 ns MD simulations were performed for each system, starting from either one of the two representative structures obtained through homology modeling (replica 1 or 2 – see above).

All Arg, Lys, Asp, and Glu residues were considered to be in ionized form. The protonation state of the His residues took into account the hydrogen bond environment. For each subunit of the dimer, H58, H168, H184 and H191 were considered Nε-protonated, while H36 was Nδ-protonated, and H19 and H29 were in doubly-protonated form (note that H29 can form a salt bridge with a phosphodiester of the RNA distal box). The assigned titration states are in agreement with the pKa values predicted by propKa[18] and with the optimized hydrogen placement algorithm of MolProbity[19]. Phosphoserine

(either pS33 or pS34) was modeled in doubly unprotonated form (*i.e.* with a –2 charge), since the first and second pKa values of the phosphomonoester group are 2.19 and 5.78, respectively (see [20] and references therein). In the diphosphorylated state (both pS33 and pS34), two possible protonation states were considered: doubly unprotonated (–2 charge) pS33 together with singly unprotonated (–1 charge) pS34 or vice versa. The Glu side chains of the S33E mutant, the S34 mutant and the S33E,S34E double mutant were considered as unprotonated (*i.e.* E33 and E34 have a –1 charge each).

The protein and the dsRNA were described using the Cornell et al.[21] and parm99bsc0[22] force fields, respectively. The TIP3P model was used for the water molecules[23] and the Åqvist parameters for the sodium and chloride ions[24]. The parameters for phosphoserine were taken from reference[25]. The metal ion-containing catalytic site of the product-bound model was treated using a flexible non-bonded approach[26]. The Mg^{2+} Lennard-Jones interactions were calculated using the metal ion van der Waals parameters of Allnér et al.[27]. The metal-ligand electrostatic interactions were computed using reparameterized charges[26], based on the "atoms in molecules" partitioning scheme[28] of the DFT-BLYP level electronic density, in order to account for metal-ligand charge transfer effects. Namely, a gas phase model of the catalytic site was built, including the conserved residues (D45, E41 and E117, *E. coli* residue numbering); the metal ions (MgB and MgA) and coordinated water molecules (W1-W4); and the two nucleotides joined by the scissile phosphodiester (see Fig. S7). The side chains of the conserved acidic residues are modeled as acetate (D45) or propionate (E41 and E117), whereas the 5' adenosine ribonucleotide at site 0 (R0) is modeled as a methylphosphate, and the 3' guanosine ribonucleotide at site -1 (R-1) as a methoxide. The 3'-oxygen was considered as unprotonated (*i.e.* a 3'-alkoxide), because the template crystal structure was obtained at basic pH (8.5). Moreover, test MD simulations showed that a protonated leaving group (*i.e.*

3'-hydroxyl) is not able to maintain coordination to the MgB catalytic metal ion, as observed in the crystal structure. The geometry of the active site model was optimized by means of Density Functional Theory (DFT), keeping fixed the positions of the C_β atoms of the acidic residues, as well as the C3' and C5' atoms of the nucleotides, in order to mimic the steric constraints imposed by the protein and the nucleic acid. The calculations were performed with the CPMD program[29], using the BLYP exchange-correlation functional, norm-conserving Martins-Troullier pseudo-potentials, and a plane wave cutoff of 70 Ry. The resulting main active site distances and Bader-corrected charges are listed in Tables S1 and S2, respectively. A similar non-bonded model of the metal-containing active site has been successfully used with other ribonucleases, such as RNase H[30,31] and the influenza PA endonuclease[32].

All simulations were performed with the NAMD program (version 2.9)[33]. The SHAKE/RATTLE algorithm[34] was used to constrain the bonds involving hydrogen atoms in both the solvent and the solute. Multiple time step integration was carried out using r-RESPA[35], with a base time step of 2 fs and a secondary time step of 4 fs for long-range interactions. Periodic boundary conditions were applied and the particle mesh Ewald (PME) method[36] was employed to evaluate long-range electrostatic interactions. All systems were simulated in the NPT ensemble, with a Nosé-Hoover thermostat[37] and a Langevin piston Nosé-Hoover barostat to maintain the temperature at 300 K and the pressure at 1 atm, respectively.

Control MD simulations

To provide a control for the homology modeling protocol, MD simulations were also performed for *Aa*-RNase III. In this regard, *Aa*-RNase III is also phosphorylated *in vitro* by T7PK (see Fig. S6), and *Aa*-S31 is equivalent to *Ec*-S33 (see Fig. S1). The assumption

here is that S31 is the site of phosphorylation in the *Aquifex* enzyme. The initial coordinates of the *Aa*-RNase III enzyme in complex with cleaved dsRNA were taken from PDB entry 2NUG[4], which is the same structure used as template in the homology modelling of *Ec*-RNase III. The *Aa*-RNase III system was set up using the same protocol applied to *Ec*-RNase III (see above). Two states were considered: (i) the wild-type, non-phosphorylated homodimer (wt–wt') and (ii) wild-type enzyme with both subunits phosphorylated at Ser31 (pS31–pS31'). Each of the two simulations was extended for 100 ns.

Interaction analysis of the MD simulations

During the MD simulations, *Ec*-RNase III residues at position 33 and 34 established interactions either with solvent molecules (unphosphorylated wt) or with residues K35 and R95 (phosphorylated wt, see Fig. 1 and Fig. S9, or the phosphomimetic mutants, see Fig. S11). We therefore further analyzed the stability of the interactions with K35 and R95 along the MD simulations for each of the simulated systems. The hydrogen bonds / salt bridges of K35 with residues 33 (S33/pS33/E33) and 34 (S34/pS34/E34) were described using the distance with the amino nitrogen atom (N_ζ), whereas for R95 two distances were used, with either one of the two guanidinium end nitrogen atoms (N_{H1} and N_{H2}), in order to accommodate the possibility of bidentate interactions. For S33 and S34, the distances were measured with respect to the hydroxyl oxygen atom (O_γ) of serine, and thus the presence of a hydrogen bond with K35 or R95 is indicated by values shorter than 3.5 Å. For pS33 and pS34, the distances were measured with respect to the phosphorus atom of phosphoserine (P), in order to include salt bridges with any of the three equivalent phosphate oxygen atoms. Similarly, for E33 and E34 the distances were measured with respect to the carboxylate carbon atom (C_δ), such that salt bridges with any of the two equivalent carboxylate oxygen atoms are taken into account. Therefore, the presence of a

salt bridge between the phosphate (pS33/pS34) or carboxylate (E33/E34) group and the basic residues (K35 or R95) is indicated by distances shorter than 4.0 Å.

Moreover, for the diphosphorylated wt systems, two additional interactions were observed. First, a salt bridge can be established between the two phosphoserines. Therefore, the distance between the phosphorus atom (P) of the (2-) pSer and the protonated oxygen of the (-1) pSer was also measured; values shorter than 4.0 Å are indicative of the presence of a salt bridge between pS33 and pS34. Second, pS34 was observed to interact temporarily with R62 of the opposite subunit (i.e. pS34-R62' or pS34'-R62 interactions). Hence, the distance between the phosphorus atom (P) of pS34 and either one of the two guanidinium end nitrogen atoms (N_{H1} and N_{H2}) of R62, was also measured; values shorter than 4.0 Å are indicative of the presence of a salt bridge.

To assess the stability of the hydrogen bonds or salt bridges during the MD simulations, histograms of the corresponding distances were constructed using a 0.1 Å bin, then normalized by the simulation time (~40 ns, since the first 10 ns were considered as equilibration and discarded). For each of the *Ec*-RNase III systems, the results of the two independent simulations, started from the two different replicas obtained upon homology modelling (Fig. S2), were analyzed separately. The resultant histograms are shown in Figs. S8, S10 and S12, and the percentage occurrence of the hydrogen bonds / salt bridges are listed in Tables S3-S4.

Interaction analysis of the control MD simulations

The MD simulations of the *Aa*-RNase III enzyme revealed interaction patterns (Fig. S13) similar to those in the *Ec*-RNase III simulations (Fig. 1). *Aa*-S31 (equivalent to *Ec*-S33, see Figs. S1 and S2) was observed to interact either with solvent molecules (unphosphorylated wt) or with *Aa*-R94 (phosphorylated wt), which is equivalent to *Ec*-R95.

We also examined *Aa*-K33 and *Aa*-K32, as either could take the place of *Ec*-K35 (see Fig. S1). In the sequence alignment of Court et al.[10], *Aa*-K33 is equivalent to *Ec*-K35, whereas, in our alignment, *Aa*-K33 is shifted by a single position (Fig. S1); the actual positioning cannot be assigned with full confidence due to the different length of the two phospholoops and their lack of structure. Moreover, in both alignments, *Aa*-K32 replaces *Ec*-S34. Therefore, we analyzed the interactions of S31 or pS31 with all three possible partners: R94, K33 and K32. Histograms of the corresponding distances were constructed using a 0.1 Å bin and then normalized by the simulation time (~90 ns, since the first 10 ns were considered as equilibration and discarded). The obtained histograms are presented in Fig. S14 and the percentage occurrences of the hydrogen bonds / salt bridges are listed in Tables S5-S6.

Bioinformatics analysis of the phospholoop.

The region between helices α_2 and α_3 of *Ec*-RNase III, representing the target of T7PK phosphorylation (here denoted the *phospholoop*), was analyzed further in terms of the number and position of serine residues. First, BLASTP[38] and the UniProt database[39] were used to search for predicted 0.7 protein sequences in other T7-like phages (note that T7PK is the N-terminal part of the 0.7 protein[40]). Ten other T7 group phages with predicted 0.7 protein orthologs were identified (Table S8). The corresponding 0.7 polypeptide sequences were aligned using Clustal Omega[41] and the alignment was visualized with ESPript[1]. The 0.7 protein orthologs have ~50% sequence identity (see Table S8), except for the ΦA318 phage protein (~32%). The glycine residue (G76 in T7PK) implicated in binding ATP[4] is highly conserved (see Fig. S15), strongly suggesting that the predicted 0.7 protein orthologs possess phosphotransferase activity. Moreover, the serine residues that are self-phosphorylated in T7PK (S216 and S218)[7] are also conserved. S216, the primary self-phosphorylation site, is fully conserved, whereas the secondary phosphosite, S218, is

conserved in nine of the eleven sequences, and is substituted by an also phosphorylatable Thr residue in the *Stenotrophomonas* T7PK ortholog.

Next, representative bacterial hosts were selected for the T7-related viruses and the corresponding RNase III sequences were retrieved (see Table S9). The polypeptide sequences were aligned using Clustal Omega[41] and visualized with ESPript[1]. The *E. coli* residues S33 and S34, identified in this work as alternative targets of T7PK phosphorylation, are conserved in most RNase III orthologs from bacteria infected by T7-like phages (see Fig. S16). Moreover, *Ec*-R95 and K35, predicted here to be involved in the phosphorylation-mediated rate enhancement, are also conserved (Fig. S16), with the only exceptions being *Stenotrophomonas* and *Vibrio*. However, it is noteworthy that these two bacterial outliers have also the two RNase III orthologs with the lowest sequence identity (47.1% and 69.0%), in contrast to values > 90% for the other RNase III sequences (Table S9). Moreover, the T7-like phages infecting *Stenotrophomonas* and *Vibrio* (IME15 and ΦA318, respectively) encode 0.7 protein orthologs with the lowest sequence identity (Table S8). It is therefore reasonable to propose that differences in the RNase III sequence result in a change of phosphorylation site, and as a consequence, relate to a variation in the phage protein kinase sequence responsible for recognizing the target.

In addition, we extended the bioinformatics analysis to a larger multiple sequence alignment (MSA) of 1,138 bacterial RNase III sequences from the rp75 sequence database (see reference [11] for details on the construction of the MSA). Sequence logos[8] were built for the phosphorylatable segment between helices α2 and α3, using Weblogo (version 3)[9]. Fig. S17 displays two sequence logos: a "full" sequence logo generated for the whole set of 1,138 RNase III sequences (in which the α2–α3 segment exhibits a variable length), and a "biased" sequence logo for a subset of 314 RNase III sequences (where the α2–α3 loops have the same length (~12 aa) as in *Ec*-RNase III). In both logos,

Ec-S33 and S34 are only partially conserved, compared to the almost full conservation in the RNase III orthologs of bacteria infected by T7PK-like-containing phages (Fig. S16). However, one must take into account the observation that phosphorylation sites are less conserved than the average protein sequence[42,43]. Phosphosites are mostly located in highly accessible and flexible regions, including loops and “hinges”, and hence there is a competition between fast sequence evolution of unstructured regions and the evolutionary constraint to preserve a functional (phosphorylatable) site. In this regard, a BLAST search did not identify a cellular kinase ortholog of the 0.7 protein, and thus there is no evident bacterial evolutionary pressure to preserve phosphorylatable serine residues in the α2–α3 segment.

Finally, it is noteworthy that there is an additional serine residue in the α2–α3 segment, *Ec*-S31, that is highly conserved (98.2% for the full set of sequences, and 100% for the subset). However, the conservation of *Ec*-S31, along with the three preceding residues (T28, H29 and R30), most likely reflects a conserved function of the THRS tetrad in dsRNA binding, rather than phosphorylation. In this regard, the crystal structure of *Aa*-RNase III in complex with a minimal size product (PDB entry 2NUG)[4] shows *Aa*-H27 (*i.e.* the residue equivalent to *Ec*-H29) interacting with the 3'-phosphodiester group of the 5' end nucleotide (see also Table S7). Moreover, *Aa*-S29 (*i.e.* the residue equivalent to *Ec*-S31) forms a hydrogen bond with *Aa*-H27[4]. We hypothesize that this hydrogen bond may stabilize the specific rotameric state of the imidazole ring necessary for optimal interaction with dsRNA. Alternatively, a modest rotation of the serine side chain would allow formation of a hydrogen bond with the ribose 2'-hydroxyl group of the 5' end adjacent nucleotide. Besides this structural data, alanine mutations of the THRS tetrad in the *A. aeolicus* enzyme (N. R. Alla and A.W.N., unpublished experiments) reveal a significant effect on the catalytic activity, especially for the histidine residue, further supporting the proposed role of

the THRS tetrad in dsRNA binding. Interestingly, the fact that the THRS tetrad and the phosphorylatable serine residue(s) are both located in the same segment suggests a general role of the α2-α3 loop in dsRNA binding and release.

References

1. Robert, X., & Gouet, P. Deciphering key features in protein structures with the new ENDscript server. *Nucleic Acids Res.* **42**, W320-W324 (2014).

2. Buchan, D. W., Minneci, F., Nugent, T. C., Bryson, K., & Jones, D. T. Scalable web services for the PSIPRED Protein Analysis Workbench. *Nucleic Acids Res.* **41**, W349-W357 (2013).

3. Gan, J. *et al*. Structural insight into the mechanism of double-stranded RNA processing by ribonuclease III. *Cell* **124**, 355-366 (2006).

4. Gan, J. *et al*. A stepwise model for double□stranded RNA processing by ribonuclease III. *Mol. Microbiol.* **67**, 143-154 (2008).

5. Amarasinghe, A.K., Calin-Jageman, I., Harmouch, A., Sun, W. & Nicholson, A.W. *Escherichia coli* ribonuclease III: affinity purification of hexahistidine-tagged enzyme and assays for substrate binding and cleavage. *Meth. Enzymol.* **342**,143–158 (2001).

6. Marchand, I., Nicholson, A. W., & Dreyfus, M. High-level autoenhanced expression of a single-copy gene in *Escherichia coli*: overproduction of bacteriophage T7 protein kinase directed by T7 late genetic elements. *Gene* **262**, 231-238 (2001).

7. Gone, S., & Nicholson, A. W. Bacteriophage T7 protein kinase: Site of inhibitory autophosphorylation, and use of dephosphorylated enzyme for efficient modification of protein *in vitro*. *Protein Expr. Purif.* **85**, 218-223 (2012).

8. Schneider, T. D., & Stephens, R. M. Sequence logos: a new way to display consensus sequences. *Nucleic Acids Res.* **18**, 6097-6100 (1990).

9. Crooks, G. E., Hon, G., Chandonia, J. M., & Brenner, S. E. WebLogo: a sequence logo generator. *Genome Res.* **14**, 1188-1190 (2004).

10. Court, D. L. *et al*. RNase III: Genetics and Function; Structure and Mechanism. *Annu. Rev. Genet.* **47**, 405-431 (2013).

11. Paudyal, S., *et al*. Combined computational and experimental analysis of a complex of ribonuclease III and the regulatory macrodomain protein YmdB. *Proteins* **83**, 459-472 (2015).

12. Baker, D., & Sali, A. Protein structure prediction and structural genomics. *Science* **294**, 93-96 (2001).

13. Eramian, D., Eswar, N., Shen, M. Y., & Sali, A. How well can the accuracy of comparative protein structure models be predicted?. *Prot. Sci.* **17**, 1881-1893 (2008).

14. Sali, A., & Blundell, T. L. Comparative protein modelling by satisfaction of spatial restraints. *J. Mol. Biol.* **234**, 779-815 (1993).

15. Lindahl, E., Hess, B., & Van Der Spoel, D. GROMACS 3.0: a package for molecular simulation and trajectory analysis. *J. Mol. Model.* **7**, 306-317 (2001).

16. Jarvis, R. A., & Patrick, E. A. Clustering using a similarity measure based on shared near neighbors. *IEEE Trans. Comput.* **100**, 1025-1034 (1973).

17. Fiser, A., Do, R. K. G., & Sali, A. Modeling of loops in protein structures. *Prot. Sci.* **9**, 1753-1773 (2000).

18. Olsson, M. H., Søndergaard, C. R., Rostkowski, M., & Jensen, J. H. PROPKA3: consistent treatment of internal and surface residues in empirical pKa predictions. *J. Chem. Theory Comput.* **7**, 525-537 (2011).

19. Davis, I. W. *et al*. MolProbity: all-atom contacts and structure validation for proteins and nucleic acids. *Nucleic Acids Res.* **35**, W375-W383 (2007).

20. Smiechowski, M. Theoretical pKa prediction of O-phosphoserine in aqueous solution. *Chem. Phys. Lett.* **501**, 123-129 (2010).

21. Cornell, W. D. *et al*. A second generation force field for the simulation of proteins, nucleic acids, and organic molecules. *J. Am. Chem. Soc.* **117**, 5179-5197 (1995).

22. Pérez, A. *et al*. Refinement of the AMBER force field for nucleic acids: improving the description of α/γ conformers. *Biophys. J.* **92**, 3817-3829 (2007).

23. Jorgensen, W. L., Chandrasekhar, J., Madura, J. D., Impey, R. W., & Klein, M. L. Comparison of simple potential functions for simulating liquid water. *J. Chem. Phys.* **79**, 926-935 (1983).

24. Aaqvist, J. Ion-water interaction potentials derived from free energy perturbation simulations. *J. Phys. Chem.* **94**, 8021-8024 (1990).

25. Homeyer, N., Horn, A. H., Lanig, H., & Sticht, H. AMBER force-field parameters for phosphorylated amino acids in different protonation states: phosphoserine, phosphothreonine, phosphotyrosine, and phosphohistidine. *J. Mol. Model.* **12**, 281-289 (2006).

26. Dal Peraro, M. *et al*. Modeling the charge distribution at metal sites in proteins for molecular dynamics simulations. *J. Struct. Biol.* **157**, 444-453 (2007).

27. Allnér, O., Nilsson, L., & Villa, A. Magnesium ion–water coordination and exchange in biomolecular simulations. *J. Chem. Theory Comput.* **8**, 1493-1502 (2012).

28. Bader, R. F. Atoms in Molecules: A Quantum Theory in *International Series of Monographs on Chemistry*, Vol. 22 (Oxford University Press, 1990).

29. CPMD consortium, *CPMD program*. Available at: http://www.cpmd.org. Copyright IBM Corp. 1990-2008, Copyright Max Planck Institute, Stuttgart 1997-2001, Copyright jointly by IBM Corp. and by MPI Stuttgart 2000-2015 (2015).

30. De Vivo, M., Dal Peraro, M., & Klein, M. L. Phosphodiester cleavage in ribonuclease H occurs via an associative two-metal-aided catalytic mechanism. *J. Am. Chem. Soc.* **130**, 10955-10962 (2008).

31. Ho, M. H., De Vivo, M., Dal Peraro, M., & Klein, M. L. Understanding the effect of magnesium ion concentration on the catalytic activity of ribonuclease H through computation: does a third metal binding site modulate endonuclease catalysis?. *J. Am. Chem. Soc.* **132**, 13702-13712 (2010).

32. Xiao, S. *et al*. Magnesium-dependent RNA binding to the PA endonuclease domain of the avian influenza polymerase. *J. Phys. Chem. B* **118**, 873-889 (2014).

33. Phillips, J. C. *et al*. Scalable molecular dynamics with NAMD. *J. Comput. Chem.* **26**, 1781-1802 (2005).

34. Ryckaert, J. P., Ciccotti, G., & Berendsen, H. J. Numerical integration of the cartesian equations of motion of a system with constraints: molecular dynamics of n-alkanes. *J. Comput. Phys.* **23**, 327-341 (1977).

35. Izaguirre, J. A., Reich, S., & Skeel, R. D. Longer time steps for molecular dynamics. *J. Chem. Phys.* **110**, 9853-9864 (1999).

36. Darden, T., York, D., & Pedersen, L. Particle mesh Ewald: An N·log (N) method for Ewald sums in large systems. *J. Chem. Phys.* **98**, 10089-10092 (1993).

37. Hoover, W. G. Canonical dynamics: equilibrium phase-space distributions. *Phys. Rev. A* **31**, 1695 (1985).

38. Altschul, S. F., Gish, W., Miller, W., Myers, E. W., & Lipman, D. J. Basic local alignment search tool. *J. Mol. Biol.* **215**, 403-410 (1990).

39. UniProt Consortium. UniProt: a hub for protein information. *Nucleic Acids Res.* **43**, D204-D212 (2015).

40. Rahmsdorf, H. J. *et al*. Protein kinase induction in *Escherichia coli* by bacteriophage T7. *Proc. Natl. Acad. Sci. U.S.A.* **71**, 586-589 (1974).

41. Sievers, F. *et al*. Fast, scalable generation of high quality protein multiple sequence alignments using Clustal Omega. *Mol. Syst. Biol.* **7**, 539 (2011).

42. Gnad, F. *et al*. PHOSIDA (phosphorylation site database): management, structural and evolutionary investigation, and prediction of phosphosites. *Genome Biol.* **8**, R250 (2007).

43. Miller, M. L. *et al*. NetPhosBac – a predictor for Ser/Thr phosphorylation sites in bacterial proteins. *Proteomics* **9**, 116-125 (2009).
